# Immunotherapy Approaches for the Treatment of Triple-Negative Breast Cancer

**DOI:** 10.3390/cancers18030464

**Published:** 2026-01-30

**Authors:** Shaimaa Alharbi, Farah Faozi Qasem, Mahsa Taleb Talebi, Nourhan E. Omran, Rifat Hamoudi, Rania Harati

**Affiliations:** 1 Department of Pharmacy Practice and Pharmacotherapeutics, College of Pharmacy, University of Sharjah, Sharjah P.O. Box 27272, United Arab Emirates; d_shaimaal7arbi@hotmail.com (S.A.); u18100307@sharjah.ac.ae (F.F.Q.); mahsat507@gmail.com (M.T.T.); u24103340@sharjah.ac.ae (N.E.O.); 2 Research Institute of Medical and Health Sciences, University of Sharjah, Sharjah P.O. Box 27272, United Arab Emirates; 3 Department of Clinical Sciences, College of Medicine, University of Sharjah, Sharjah P.O. Box 27272, United Arab Emirates; 4 BIMAI (Biomedically-InforMed Artificial Intelligence) Laboratory, University of Sharjah, Sharjah P.O. Box 27272, United Arab Emirates; 5 Center of Excellence for Precision Medicine, Research Institute of Medical and Health Sciences, University of Sharjah, Sharjah P.O. Box 27272, United Arab Emirates; 6 Division of Surgery and Interventional Science, University College London, London NW3 2PF, UK; 7 ASPIRE Precision Medicine Research Institute Abu Dhabi, University of Sharjah, Sharjah P.O. Box 27272, United Arab Emirates

**Keywords:** triple-negative breast cancer (TNBC), immunotherapy, immune checkpoint inhibitors (ICIs), PD-1/PD-L1, tumor-infiltrating lymphocytes (TILs), adoptive cell therapy, CAR-T cells, oncolytic virotherapy, antibody–drug conjugates, tumor microenvironment, immune resistance

## Abstract

Triple-negative breast cancer (TNBC) is an aggressive subtype of breast cancer characterized by high immunogenicity and specific immune signatures. Although these molecular features including elevated tumor-infiltrating lymphocytes (TILs) and programmed death-ligand 1 (PD-L1) expression provide a strong rationale for immunotherapy, clinical response remains limited due to multiple mechanisms of immune escape. This review summarizes current and emerging immunotherapeutic strategies in TNBC, including immune checkpoint inhibitors (PDL-1 and cytotoxic T-lymphocyte-associated protein 4 (CTL-4) blockade), adoptive cell therapy (ACT) (chimeric antigen receptor T-cell therapy (CAR-T) and TIL therapy), oncolytic virotherapy, and antibody-based approaches. We also discuss the mechanisms of resistance including DNA damage response alterations, anti-apoptotic signaling, and tumor microenvironment-mediated resistance. Finally, we highlight rational combination strategies, immunotherapy with chemotherapy, targeted therapy, or additional immunotherapies that aim to enhance response to immunotherapy. Ongoing advances in immunotherapy hold significant potential to improve outcomes for patients with TNBC.

## Simple Summary

Triple-negative breast cancer (TNBC) is an aggressive breast cancer subtype with limited treatment options and poor clinical outcomes. Despite its relatively high immunogenicity, responses to immunotherapy remain restricted to a subset of patients. This narrative review provides a comprehensive overview of current and emerging immunotherapeutic approaches for TNBC including immune checkpoint inhibition, adoptive cell therapies, oncolytic viruses, and antibody-based strategies. We also summarize key molecular and cellular mechanisms that limit therapeutic efficacy such as alterations in DNA damage response pathways, activation of anti-apoptotic signaling and immunosuppressive features of the tumor microenvironment. Finally, we discuss rational combination strategies designed to enhance antitumor immune responses. Ongoing advances in immunotherapy hold significant potential to improve outcomes for patients with TNBC.

## 1. Introduction

Breast cancer is the most common malignancy diagnosed among women worldwide and represents a biologically heterogeneous disease composed of several molecular subtypes with distinct prognostic and therapeutic implications [1,2]. These subtypes are broadly classified according to the expression of estrogen receptor (ER), progesterone receptor (PR), and human epidermal growth factor receptor 2 (HER2) resulting in hormone receptor-positive, HER2-positive, and triple-negative breast cancer (TNBC) categories [3].

TNBC accounts for approximately 10–15% of all breast cancer cases and is recognized as the most aggressive and clinically challenging subtype due to the absence of ER, PR, and HER2 expression, which eliminates the use of endocrine and HER2-targeted therapies [4]. As a result, cytotoxic chemotherapy, constituted primarily by anthracyclines, taxanes, and platinum agents, has for many years been the cornerstone of management for both early-stage and mTNBC (mTNBC) [3]. However, despite initial responses, chemotherapy is associated with limited durability and outcomes remain particularly poor in relapsed or metastatic disease, underscoring the urgent need for more effective treatment strategies [5].

A key feature that distinguishes TNBC from other breast cancer subtypes is its relatively high immunogenicity. Elevated levels of tumor-infiltrating lymphocytes (TILs), increased programmed death-ligand 1 (PD-L1) expression, and higher tumor mutational burden (TMB) suggest that the immune system plays a central role in TNBC biology [6,7]. These characteristics provide a strong biological rationale for exploring immunotherapy as a therapeutic strategy in this disease. Consequently, immunotherapy has rapidly emerged as a promising approach to improve outcomes in TNBC with multiple agents and combination strategies now under active investigation. However, many TNBC patients still do not respond to monoimmunotherapy, with response remaining low at approximately 15–20% [8].

Immunotherapy is a transformative therapeutic approach in oncology that aims to enhance the body’s antitumor immune response while minimizing systemic toxicity either by stimulating immune effector cells or by removing inhibitory signals within the tumor microenvironment (TME) [9]. It has demonstrated remarkable success across multiple solid tumors in recent years, showing the potential not only to control disease but, in some cases, to achieve durable remission. Several classes of immunotherapeutic strategies are currently clinically established, including immune checkpoint inhibitors (ICIs), which are now the standard of care, in combination with chemotherapy, for high-risk early-stage TNBC, while others, such as adoptive cell therapy (ACT), monoclonal antibodies (mABs), and oncolytic viruses (OVs), remain under investigation [10,11,12,13].

In this narrative review, we provide a comprehensive and structured overview of the current landscape of immunotherapy in TNBC. We discuss the immunogenic features that distinguish TNBC from other breast cancer subtypes, summarize the mechanisms and clinical evidence supporting the use of ICIs, and explore emerging immunotherapeutic modalities. Furthermore, we examine mechanisms of therapeutic resistance, highlight ongoing efforts to optimize combination strategies, and outline future directions that may guide the development of more effective immune-based approaches for TNBC.

## 2. Immunogenic Landscape of Triple-Negative Breast Cancer

Several studies have demonstrated that TNBC tumors are immunologically active, displaying features associated with improved responsiveness to immune-based treatments. One of the key characteristics of TNBC is the presence of high levels of TILs. Elevated TIL density has been correlated with improved pathological complete response (pCR) rates following chemotherapy and better overall survival (OS), suggesting a pre-existing antitumor immune response within the TME [14].

TNBC also shows higher expression of PD-L1 on tumor and immune cells compared with hormone receptor-positive breast cancers [15]. Increased PD-L1 expression is clinically relevant because it predicts response to ICIs targeting the programmed cell death protein (PD-1)/PD-L1 axis in mTNBC, where PD-L1 status is used to guide treatment selection [16,17]. However, recent data suggest that PD-L1 immunohistochemistry (IHC) alone may not reliably predict benefit [18,19,20]. Instead, combined immune biomarkers including TIL density, a T-cell inflamed gene expression profile (GEP), and TMB provide more robust prediction of the response to ICI therapy in TNBC [21]. In the neoadjuvant setting, clinical benefit from chemo-immunotherapy has been observed irrespective of PD-L1 expression, indicating that PD-L1 is not a reliable predictive biomarker in early-stage disease [19,22,23].

Another important immunogenic feature of TNBC is its TMB. Approximately 10–20% of TNBC patients harbor germline or somatic breast cancer susceptibility gene 1 and 2 (BRCA1/2) mutations or exhibit homologous recombination deficiency (HRD), which contributes to genomic instability, TMB, and increased neoantigen load [24,25]. While high TMB increases the likelihood of neoantigen generation and may enhance tumor visibility to the immune system, the TMB of TNBC likely contributes to immunogenicity but does not independently determine responsiveness to immune checkpoint blockade (ICB) [26]. Indeed, absolute TMB levels in TNBC are generally modest, typically ranging from approximately 1.5 to 3 mutations/megabase (Mb), which is below the established high-TMB thresholds (≥10 mutations/Mb) used for ICI response prediction across tumor types [6,27,28,29].

Additionally, the TNBC microenvironment often contains activated cytotoxic T cells, dendritic cells (DCs), and pro-inflammatory cytokines, reflecting an overall “inflamed” or “immunologically hot” tumor phenotype [30]. This phenotype supports antigen presentation and T-cell infiltration, both of which are prerequisites for successful immunotherapy. However, TNBC also employs multiple immunosuppressive pathways such as upregulation of PD-L1, recruitment of myeloid-derived suppressor cells (MDSCs), and expansion of regulatory T cells (Tregs), which collectively limit the effectiveness of spontaneous immune responses and create opportunities for therapeutic intervention [31,32]. Together, these immunogenic features provide strong justification for the clinical exploration of immunotherapy in TNBC.

## 3. Immunotherapy Approaches in Triple Negative Breast Cancer

Immunotherapy has emerged as a promising therapeutic strategy for TNBC. This section provides an overview of the major immunotherapeutic modalities that have been evaluated in TNBC, including ICIs, ACT, OVs, and mABs.

### 3.1. Immune Checkpoints Inhibitors in Triple Negative Breast Cancer

**Figure 1:**
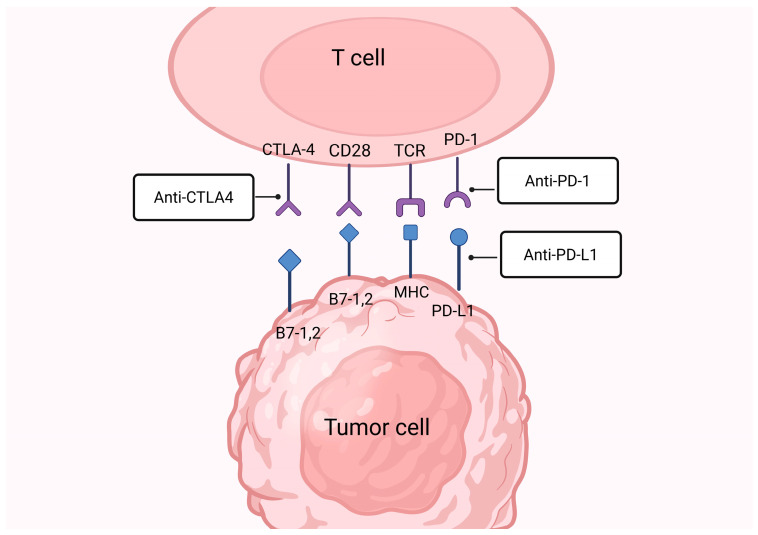
Mechanisms of immune checkpoint inhibition in TNBC. This figure illustrates the major inhibitory immune checkpoint pathways that regulate T-cell activity at distinct stages of the antitumor immune response. CTLA-4 primarily functions during the early T-cell priming phase in secondary lymphoid organs, where it competitively binds B7-1 (CD80) and B7-2 (CD86) on antigen-presenting cells with higher affinity than the co-stimulatory receptor CD28, thereby limiting initial T-cell activation. PD-1/PD-L1 signaling predominantly operates during the effector phase within the TME. Tumor and immune cells express PD-L1, which binds PD-1 on activated T cells, leading to T-cell exhaustion and reduced cytotoxic function. ICIs targeting PD-1, PD-L1, or CTLA-4 block these interactions, restore T-cell activation, and enhance antitumor responses [36]. Created using BioRender.com.

Immune checkpoints comprise proteins expressed on the surface of immune cells that regulate the immune responses and help in preventing auto-immune reactions [33]. These molecules are broadly classified in two functional groups, co-stimulatory checkpoints such as cluster of differentiation 27 (CD27), CD28, CD137, inducible T-cell co-stimulator (ICOS), CD137 (4-1BB), and CD134 (OX-40), which promote T-cell activation and survival, and co-inhibitory checkpoint including the cytotoxic T-lymphocyte-associated protein 4 (CTLA-4), PD-1, lymphocyte activation gene 3 (LAG-3), T-cell immunoglobulin and mucin-domain containing-3 (TIM-3), T-cell immunoreceptor with Ig and immunoreceptor tyrosine-based inhibitory motif (ITIM) domains (TIGIT), and V-domain Ig suppressor of T-cell activation (VISTA), which reduce T-cell activity to limit excessive inflammation and autoimmunity [34,35]. Immune checkpoint pathways regulate T-cell responses at distinct stages of the antitumor immune response. CTLA-4 primarily functions during the early “priming” phase in secondary lymphoid organs, where it competes with CD28 for binding to B7 molecules (B7-1/CD80 and B7-2/CD86) on antigen-presenting cells, thereby limiting initial T-cell activation [36]. In contrast, PD-1/PD-L1 signaling predominantly operates during the “effector” phase within the TME, where engagement of PD-1 on activated T cells by PD-L1 expressed on tumor cells and immune cells suppresses cytotoxic T-cell function and promotes immune evasion (Figure 1) [37].

Within the TME, inhibitory checkpoint pathways are frequently upregulated [38]. Their ligands, most notably PD-L1 and PD-L2, are expressed on tumor cells and various immunosuppressive stromal cells [39]. Interaction of these ligands with the inhibitory receptors on T cells leads to reduced cytotoxic activity of T cells and enables tumor cells to evade immune surveillance [40,41]. Many cancers, including TNBC, exploit these pathways by overexpressing checkpoint ligands [42]. Consequently, therapeutic blockade of inhibitory immune checkpoints has emerged as a promising strategy to restore T-cell activation and enhance anti-tumor immunity in TNBC [37].

#### 3.1.1. PD-1 and PD-L1 Inhibitors

PD-1 is an inhibitory receptor expressed on activated T cells, B cells, monocytes, DCs, and TILs [43,44]. Under physiological conditions, the PD-1/PD-L1 interaction limits immune-mediated tissue damage and prevents autoimmunity [45]. In the TME, however, many cancers, including TNBC, upregulate PD-L1 on the surface of tumor cells and immunosuppressive stromal cells [46]. Binding of PD-L1 to PD-1 on CD8^+^ T cells induces T-cell exhaustion, reduces cytokine production, and suppresses cytotoxic activity, ultimately enabling tumor cells to evade immune surveillance [47].

Therapeutic blockade of the PD-1/PD-L1 axis can restore the activity of tumor-infiltrating T cells and re-establish antitumor immune activity [48]. ICIs targeting these pathways include PD-1 inhibitors such as pembrolizumab, which is currently approved and used in combination with chemotherapy in both early-stage and mTNBC, as well as PD-L1 inhibitors such as atezolizumab [17,49,50]. While atezolizumab demonstrated clinical benefit in early-stage TNBC in the neoadjuvant IMpassion031 trial (NCT03197935), its accelerated approval for PD-L1-positive invasive TNBC was withdrawn after the phase III IMpassion131 trial (NCT03125902) failed to confirm a survival benefit [17,49,50]. TNBC is particularly suited for ICB as it demonstrates higher PD-L1 expression and greater immune infiltration than other breast cancer subtypes [51]. In particular, the phase III KEYNOTE-355 clinical trial (NCT02819518) demonstrated that combining pembrolizumab with chemotherapy significantly improved both progression-free survival (PFS) and OS in PD-L1–positive mTNBC (combined positive score (CPS) ≥ 10) [52]. Consistent with this, clinical studies have shown that selected TNBC populations benefit from PD-1/PD-L1 inhibition [53]. More recently, a 2025 meta-analysis showed that PD-1/PD-L1 inhibitors combined with chemotherapy improve survival outcomes compared to chemotherapy alone [54]. In the phase III KEYNOTE-522 trial (NCT03036488), addition of the neoadjuvant pembrolizumab plus chemotherapy followed by administration of adjuvant pembrolizumab resulted in a significant improvement in OS among patients with early-stage TNBC as compared with neoadjuvant chemotherapy alone [55,56]. These findings implicate ICB as a cornerstone of systemic therapy in selected TNBC populations [57].

##### Pembrolizumab Monotherapy

Pembrolizumab is a humanized IgG4 mAB that targets the PD-1 receptor on T cells, thereby blocking its interaction with PD-L1 and restoring antitumor T-cell activity [58]. The first evidence supporting pembrolizumab monotherapy in TNBC emerged from the phase Ib KEYNOTE-012 trial (NCT01848834), in which pembrolizumab demonstrated an objective response rate (ORR) of 18.5% in patients with PD-L1-positive mTNBC, leading to the first regulatory approval of a PD-1 inhibitor in this subtype [59]. However, the Food and Drug Administration (FDA) indication for pembrolizumab as a monotherapy was later withdrawn for unselected mTNBC populations following subsequent phase III data that failed to show a significant survival advantage over chemotherapy [60]. Further evaluation of pembrolizumab monotherapy was conducted in the phase II KEYNOTE-086 study (NCT02447003), which assessed the dosage of 200 mg pembrolizumab every three weeks in two mTNBC cohorts. The first cohort included previously treated unselected patients and demonstrated an ORR of 5.3%, indicating limited activity in the later-line setting. The second cohort consisted of treatment-naïve patients with PD-L1-positive tumors and showed a substantially higher ORR of 21.4%, suggesting that pembrolizumab monotherapy is more effective as a first-line therapy in PD-L1-expressing mTNBC [61,62]. The phase III KEYNOTE-119 trial (NCT02555657) compared pembrolizumab with chemotherapy in previously treated PD-L1-positive mTNBC patients [63]. A 2024 follow-up analysis showed that any monotherapy benefit signal remains restricted to tumors with high PD-L1 expression (CPS ≥ 10) and without extensive immunosuppressive TME features [64]. New studies suggest that pembrolizumab monotherapy is now considered clinically insufficient outside biomarker-enriched first-line disease with high expression of PD-L1, and ongoing trials prioritize escalation to combination strategies instead [61,65]. Overall, these findings indicate that pembrolizumab monotherapy provides limited benefit in pretreated mTNBC. Specifically, its clinical activity appears to be primarily confined to PD-L1-positive tumors in the first-line metastatic setting.

##### Atezolizumab and Avelumab Monotherapy

Atezolizumab and avelumab are humanized IgG1 mABs that target PD-L1, preventing its interaction with PD-1 on T cells and thereby restoring antitumor immune activity [13]. By blocking the PD-1/PD-L1 immune checkpoint inhibitory pathway, these agents enhance T-cell activation and reduce tumor immune evasion.

The clinical activity of atezolizumab monotherapy in mTNBC was first evaluated in a multi-cohort phase I study (PCD4989g trial) (NCT01375842). In this trial, patients treated in the first-line metastatic setting achieved an ORR of 24%, whereas those treated in later lines demonstrated a markedly lower ORR of 6%. Responses were enriched in tumors with high PD-L1 expression, while PD-L1-negative tumors showed minimal or no clinical benefit, underscoring the importance of PD-L1 as a predictive biomarker [59].

Avelumab was assessed in the phase Ib JAVELIN solid tumor trial (NCT01772004), which included an unselected cohort of metastatic breast cancer patients among whom a subset had mTNBC. The ORR in the TNBC subgroup was 5.2%. Similar to findings with atezolizumab, higher response rates were observed in PD-L1-positive tumors compared with PD-L1-negative disease (22.2% versus 2.6%) [66]. Taken together, these studies show that PD-L1 inhibitors as a monotherapy exhibit modest antitumor activity in mTNBC with meaningful responses largely restricted to first-line treatment and PD-L1-enriched tumors. As single agents, their efficacy remains limited, supporting the development of combination strategies to enhance clinical benefit in this aggressive subtype. In the current clinical landscape (2023–2025), monotherapy with PD-L1 inhibitors in mTNBC is rarely adopted. Most ongoing and approved regimens incorporate PD-L1 blockade in combination with chemotherapy or other agents to improve therapeutic efficacy [58]. Table 1 provides a descriptive overview of key monotherapy trials. It is important to note that the results presented in this review represent a descriptive, secondary interpretation of published data. Statistical results are reported as presented in the original studies without independent analyses or cross-trial comparisons.

##### Biomarkers for Predicting the Efficacy of Anti-PD-1/PD-L1 Immunotherapy

Identifying reliable biomarkers is essential for determining which patients are most likely to benefit from ICI therapy and for avoiding unnecessary toxicity and financial burden in non-responders. Several biomarkers have emerged as potential predictors of response to anti-PD-1/PD-L1 therapy in TNBC.

PD-L1 Expression Level

PD-L1 is one of the most widely studied biomarkers for predicting response to anti-PD-1/PD-L1 therapy [13]. TNBC displays higher PD-L1 expression than other breast cancer subtypes, with reported positivity ranging from 21% to 56% [68]. PD-L1 is expressed predominantly on tumor-infiltrating immune cells and, to a lesser extent, on tumor cells, both of which are relevant for therapeutic response [69]. Consistent with this, multiple clinical trials have demonstrated that patients with PD-L1-positive TNBC derive greater benefit from PD-1/PD-L1 inhibitors [70,71]. However, PD-L1 is an imperfect biomarker. A subset of PD-L1-negative patients still respond to ICIs, indicating that PD-L1 expression alone cannot fully predict the therapeutic benefit [72]. Variability in PD-L1 expression across different cellular compartments within the TME, particularly on non-tumor immune cells, may contribute to these inconsistencies and limit its reliability as an exclusionary biomarker [73]. Nevertheless, the reliability of PD-L1 as a predictive biomarker is challenged by methodological and pre-analytical factors. Notably, different IHC assays and scoring systems yield variable PD-L1 positivity rates and recent data show that long-term storage of paraffin-embedded breast cancer specimens may degrade PD-L1 antigenicity, potentially leading to false-negative results and underestimation of PD-L1 prevalence in studies [74]. Importantly, reported PD-L1 positivity rates in TNBC are highly assay- and scoring-dependent. Distinct scoring algorithms including tumor proportion score, combined positive score, and immune cell (IC) scoring apply fundamentally different cutoffs and evaluate different cellular compartments, limiting direct comparability across clinical trials. In particular, the SP142 assay used in the IMpassion130 trial (NCT02425891), which relies on immune cell scoring, has been shown to systematically underestimate PD-L1 expression compared with the 22C3 and SP263 assays [75]. This assay discordance compromises cross-study interpretation and highlights the need for assay-specific context when assessing the predictive utility of PD-L1 in TNBC [76]. Finally, it is increasingly recognized that PD-L1 IHC alone provides an incomplete representation of tumor immune competence in TNBC. The field is therefore shifting toward automated digital pathology approaches that enable objective quantification of PD-L1 expression and TIL density [77].

Tumor-Infiltrating Lymphocytes

TNBC is characterized by the highest levels of TILs among all breast cancer subtypes [78]. TILs are lymphocytes present within the tumor or surrounding stroma and their presence reflects an ongoing endogenous immune response against the tumor [79]. TILs are commonly classified as stromal TILs (sTILs), located within the tumor stroma, and intratumoral TILs (iTILs), found in direct contact with tumor cells. Among these, sTILs are the primary compartment used for clinical scoring and prognostic assessment in TNBC [80,81]. The elevated sTIL levels observed in TNBC are partly attributed to its increased genomic instability, which results in a higher neoantigen load and contributes to the immunogenic nature of this subtype [82]. High sTIL density has consistently been associated with improved clinical outcomes in early-stage TNBC including higher rates of pCR and better OS, establishing sTILs as a strong prognostic indicator [18,83,84]. It should be noted that the prognostic and predictive value of TILs is highly dependent on standardized assessment methodology. According to the International Immuno-Oncology Biomarker Working Group (IIOBWG) guidelines, stromal TILs should be quantified as the percentage of stromal area occupied by mononuclear inflammatory cells on hematoxylin and eosin-stained sections [14]. Assessment is typically performed at 20× magnification using representative tumor regions while avoiding areas of necrosis or artifacts [14]. Adherence to these standardized criteria has been shown to improve reproducibility across studies and is essential for ensuring consistent prognostic associations of sTIL density in TNBC [85].

Moreover, emerging evidence suggests that sTIL levels may also predict response to ICIs. In the IMpassion130 trial evaluating atezolizumab plus nab-paclitaxel in advanced or mTNBC (NCT02425891), patients with both high sTIL levels and PD-L1 expression achieved significantly longer PFS and OS [86]. Similar findings were reported in the KEYNOTE-173 study (NCT02622074), where elevated sTILs and PD-L1 expression correlated with higher pCR rates in patients receiving pembrolizumab combined with chemotherapy [55]. Recent large-cohort analyses confirm that high sTILs levels remain strongly correlated with better pCR, disease-free survival (DFS) and OS in TNBC [83]. Additional data from mTNBC patients treated with ICIs show that high pre-treatment sTIL density or T-cell–inflamed gene signatures correlate with improved response and survival [62]. Collectively, these observations highlight TILs as a potential predictive biomarker for identifying TNBC patients who are more likely to benefit from immunotherapy.

It should be acknowledged that, while recent clinical trials largely adhere to IIOBWG guidelines, earlier studies employed heterogeneous scoring approaches, warranting cautious interpretation. Nonetheless, consistent associations between high stromal TIL density and favorable clinical outcomes support the prognostic and predictive relevance of sTILs in TNBC.

Tumor Mutational Burden

TMB defined as the total number of somatic mutations per Mb of tumor DNA has emerged as a potential biomarker for predicting response to ICIs [87]. Tumors with high TMB accumulate a larger number of non-synonymous mutations, leading to the generation of neoantigens that can be recognized by the immune system as foreign epitopes [26]. The increased availability of neoantigens enhances tumor immunogenicity and promotes stronger T-cell-mediated antitumor responses [88]. Approximately 10–20% of TNBC patients harbor germline or somatic BRCA1/2 mutations or exhibit HRD, which contributes to genomic instability, elevated TMB, and increased neoantigen load [89]. These molecular features may partly explain the enhanced immunogenicity observed in a subset of TNBC tumors. Furthermore, studies suggest that TNBC patients with high TMB (≥10 mutations/Mb) experience improved PFS when treated with ICIs, supporting the role of TMB as a predictive biomarker for checkpoint blockade in this subgroup [90]. Although TMB alone is not sufficient to determine therapeutic response, its association with enhanced neoantigen load underscores its potential value in identifying TNBC patients who are more likely to respond to anti-PD-1/PD-L1 therapy [18].

Microsatellite Instability and Mismatch Repair Deficiency

Microsatellite instability (MSI) is a hypermutable phenotype that arises from defects in the DNA mismatch repair (MMR) system [91]. Tumors with high MSI status accumulate numerous insertion-deletion mutations within microsatellite regions, resulting in increased mutational load and the generation of immunogenic neoantigens [40,69,70]. These features contribute to enhanced immune recognition and may explain the greater clinical benefit of ICIs observed in MMR-deficient cancers compared with MMR-proficient tumors [92]. Although MSI-high status is relatively rare in breast cancer, including TNBC, being reported in approximately 0.2–1.5% of cases, its biological consequences parallel those of TMB [93]. Both MSI and elevated TMB increase neoantigen formation, which may sensitize tumors to PD-1/PD-L1 blockade [94]. Therefore, MSI status, particularly when considered alongside TMB, may serve as a potential predictive biomarker to identify a small subset of TNBC patients who could derive meaningful benefit from ICI therapy [95].

#### 3.1.2. CTLA-4 Inhibitors

CTLA-4 is an inhibitory immune checkpoint receptor belonging to the immunoglobulin superfamily primarily expressed on activated T cells and Tregs [96]. CTLA-4 shares the same ligands, B7-1 (CD80) and B7-2 (CD86) with the co-stimulatory receptor CD28 but binds them with markedly higher affinity (Figure 1) [97]. This competitive binding limits CD28-mediated co-stimulation and suppresses the second signal required for full T-cell activation, thereby reducing interleukin-2 (IL-2) production and limiting T-cell proliferation [98]. As an early checkpoint in the immune response, CTLA-4 plays a key role in modulating T-cell priming and maintaining immune homeostasis [99]. Although CTLA-4 contributes to immunosuppression within the TME, its role in TNBC appears less dominant than that of the PD-1/PD-L1 axis [17]. Nevertheless, CTLA-4 signaling can impair effective T-cell priming and reduce antitumor immunity, providing a rationale for therapeutic blockade [100]. Several early-phase exploratory clinical studies evaluating anti-CTLA-4 antibodies, primarily in combination with PD-1/PD-L1 inhibitors, have been initiated in TNBC, although no phase II or III clinical efficacy data are currently available [47,101,102].

#### 3.1.3. Side Effects of Immune Checkpoint Inhibitors

ICIs can lead to immune-mediated toxicities because they enhance T-cell activation and diminish self-tolerance [103]. Although ICIs have significantly improved cancer outcomes, including in TNBC, their mechanism of action predisposes patients to a unique spectrum of autoimmune-like adverse events known as immune-related adverse events (irAEs) [104]. These reactions may involve virtually any organ system with commonly affected sites including the skin, gastrointestinal tract, endocrine glands, lungs, liver, and, less frequently, the nervous and ocular systems [105]. Ocular irAEs such as dry eye and uveitis occur infrequently but illustrate the broad range of potential immune-mediated toxicities [106]. Because ICIs are relatively new in routine oncology practice, many clinicians have limited experience in recognizing and managing irAEs, which underscores the importance of early detection, multidisciplinary care, and adherence to established management algorithms to ensure both patient safety and treatment efficacy [107]. Table 2 summarizes irAEs associated with ICIs [108].

### 3.2. Adoptive Cell Therapy in Triple Negative Breast Cancer

ACT is an emerging immunotherapeutic strategy that enhances the patient’s own immune system by expanding or genetically modifying antitumor immune cells ex vivo before reinfusion [109]. Traditional cancer treatments including chemotherapy, surgery, and radiotherapy often fail to achieve durable remission in many solid tumors and recurrence remains common despite initial responses [110]. Moreover, these modalities are frequently associated with significant toxicity that negatively affects patient quality of life [111]. The development of immunotherapy has transformed the therapeutic landscape by enabling more targeted and sustained antitumor responses with improved tolerability [112]. Among these advances, ACT has gained considerable attention due to its ability to generate robust tumor-specific immune responses [113]. Preclinical and early clinical investigations demonstrate that ACT holds substantial promise in TNBC, making it an attractive candidate for adoptive cellular approaches [114]. Several ACT modalities are currently under investigation in TNBC including chimeric antigen receptor T cell (CAR-T) and TIL therapy.

#### 3.2.1. Chimeric Antigen Receptor T Cell in Triple Negative Breast Cancer

**Figure 2:**
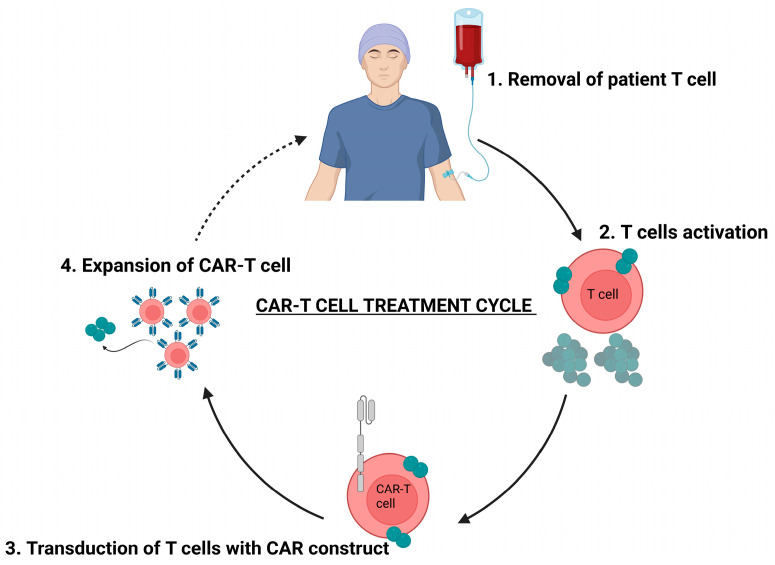
CAR-T cell therapy workflow. This figure illustrates the standard workflow of CAR-T therapy. Peripheral blood T cells are first collected from the patient through leukapheresis (step 1). Isolated T cells are subsequently activated ex vivo to promote proliferation and enhance susceptibility to genetic modification (step 2). Activated T cells are then transduced with a CAR construct—typically using viral vectors—to enable expression of a synthetic antigen-recognition receptor on their surface (step 3). The engineered CAR-T cells are expanded to clinically effective numbers before being reinfused into the patient to mediate tumor-specific cytotoxic activity (step 4). This process constitutes the complete CAR-T cell treatment cycle [116]. Created using BioRender.com.

CAR-T therapy is an ACT in which patient T lymphocytes are collected, activated, and genetically engineered ex vivo to express a synthetic receptor that recognizes tumor-associated antigens (TAAs) [115]. CAR-T manufacturing typically begins with leukapheresis to obtain peripheral blood mononuclear cells, followed by isolation and activation of T cells, which are then transduced commonly via viral vectors to introduce the CAR construct [116]. After expansion to therapeutic numbers, CAR-T cells are reinfused into the patient usually after lymphodepleting chemotherapy to enhance their engraftment and persistence (Figure 2) [117].

**Figure 3:**
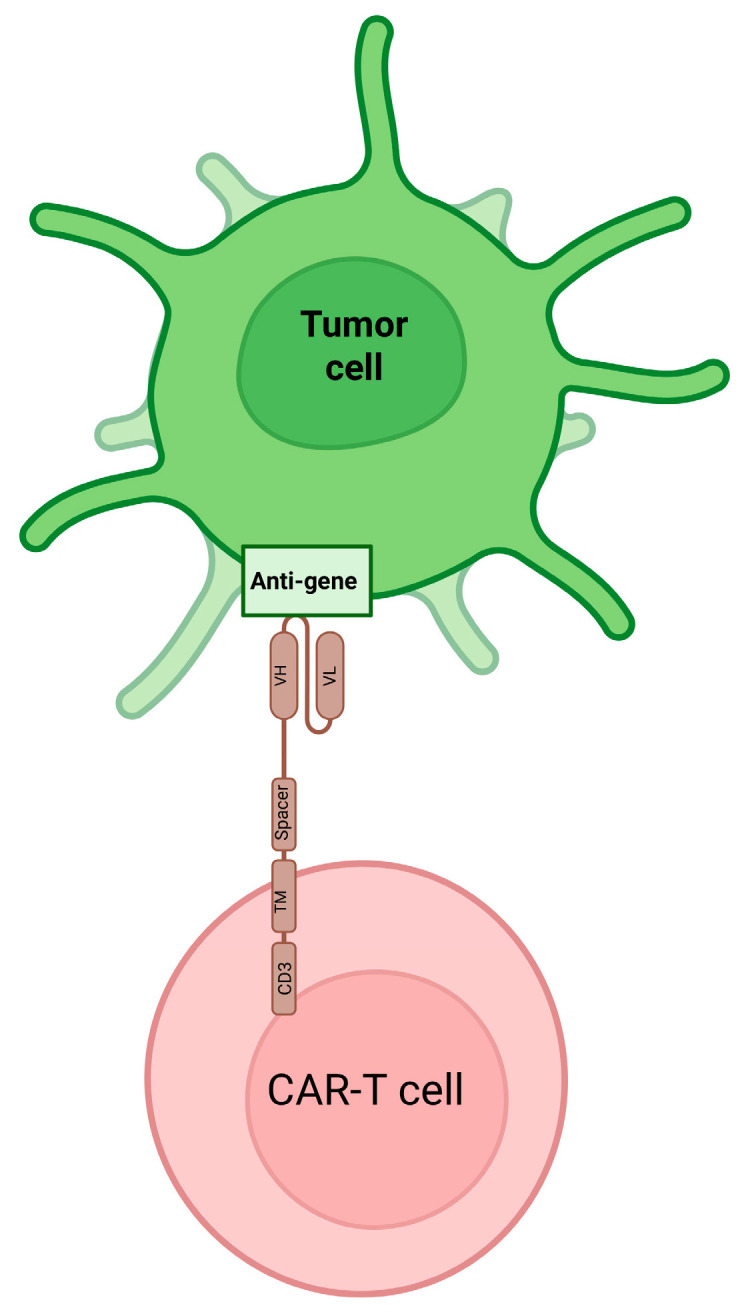
Schematic representation of CAR-T cell recognition and activation against tumor cells. This figure illustrates the mechanism of action of a CAR expressed on engineered T cells. The extracellular scFv, composed of variable heavy (VH) and variable light (VL) domains, recognizes and binds a specific TAA on the surface of the cancer cell. This binding triggers intracellular signaling through the transmembrane (TM) region and CD3ζ signaling domain, which together activate the CAR-T cell. Upon activation, the CAR-T cell exerts cytotoxic effects against the tumor cell, leading to targeted tumor cell lysis [120]. Created using BioRender.com.

A CAR molecule contains three essential components, an extracellular antigen-binding domain, typically a single-chain variable fragment (scFv) derived from mABs, a hinge and transmembrane domain that influence CAR expression and signaling strength, and an intracellular signaling domain composed of CD3ζ with one or more co-stimulatory modules [118]. Advances in CAR design, from first-generation CARs containing only CD3ζ to second- and third-generation CARs that incorporate co-stimulatory domains such as CD28, 4-1BB, OX40, or ICOS, have substantially improved T-cell activation, persistence, and cytotoxicity (Figure 3) [119].

Importantly, CAR-T cells recognize target antigens independently of major histocompatibility complex (MHC) presentation, a key advantage in solid tumors such as TNBC, where loss of MHC expression is a common immune-evasion mechanism [121]. Fourth-generation “armored” CAR-T cells (so called T cells redirected for universal cytokine killing (TRUCKs)) further enhance antitumor activity by delivering cytokines such as IL-12 directly within the TME, improving local immune activation while minimizing systemic toxicity [122]. Although still experimental in TNBC, these next-generation CAR-T strategies hold promise for overcoming barriers such as heterogeneous antigen expression, immunosuppression within the TME, and limited T-cell persistence, representing major challenges in the application of CAR-T therapy to solid tumors [123].

##### Target Antigens Investigated for CAR-T Therapy in Triple Negative Breast Cancer

A major challenge in applying CAR-T therapy to solid tumors including TNBC is the identification of TAAs that are highly expressed on cancer cells but minimally expressed in normal tissues. Several promising antigens have been explored as potential CAR-T targets in TNBC.

Chondroitin Sulphate Proteoglycan 4

Chondroitin sulphate proteoglycan 4 (CSPG4) is a highly glycosylated transmembrane proteoglycan with limited expression in normal tissues but elevated expression in several malignancies, including TNBC [124]. Preclinical studies demonstrated that CSPG4-expressing TNBC cell lines such as MDA-MB-231 and Hs578T can be effectively targeted using CSPG4-specific CAR constructs [125]. One approach involved a scFv linked to the inhibitory Tau protein, which significantly reduced TNBC cell viability, supporting CSPG4 as a potential therapeutic target [126]. To date, CSPG4-directed CAR-T strategies in TNBC remain at the preclinical stage.

Intercellular Adhesion Molecule-1

Intercellular adhesion molecule 1 (ICAM-1) is a cell surface glycoprotein involved in leukocyte adhesion and trans-endothelial migration [127]. Studies by Guo et al. showed that ICAM-1 is upregulated in TNBC cell lines and clinical tumor samples, suggesting its relevance as a biomarker and therapeutic target [128]. Furthermore, CAR-T cells directed against ICAM-1 have demonstrated cytotoxicity in vitro and represent a promising strategy for TNBC immunotherapy [128]. However, ICAM-1-targeted CAR-T approaches in TNBC have thus far been limited to preclinical evaluation.

Natural Killer Group 2D

Natural killer group 2D (NKG2D) ligands are stress-induced molecules often upregulated on tumor cells including TNBC [129]. CAR-T cells engineered to express the NKG2D receptor (NKG2DL-CAR-T cells) have shown potent antitumor activity in preclinical TNBC models [130]. A phase I clinical trial (NCT05302037) evaluating the safety and tolerability of NKG2DL-CAR-T cells in patients with solid tumors is underway [131].

Different additional TAAs have been explored as potential targets for CAR-T cell therapy in TNBC. These include epidermal growth factor receptor (EGFR), mesothelin, mucin-1 (MUC1), AXL receptor tyrosine kinase (AXL), tumor endothelial marker 8 (TEM8), integrin αvβ3, receptor tyrosine kinase-like orphan receptor 1 (ROR1), c-Met, folate receptor-α (FRα), disialoganglioside (GD2), and trophoblast cell-surface antigen 2 (TROP2) [131]. Recently, novel dual-target CAR-T strategies have been developed to overcome antigen heterogeneity in TNBC. For instance, a bispecific CAR-T targeting mesothelin (MSLN) and secreting an NKG2D–bispecific T-cell engager (BiTE) showed potent antitumor efficacy in TNBC preclinical models [130,132]. Among these candidates, mesothelin has shown particular promise due to its high tumor-restricted expression and advancing clinical development, distinguishing it from targets that remain largely preclinical [130,132].

B7-H3 (CD276)

B7-H3 (CD276) is an immune checkpoint molecule frequently overexpressed in solid tumors including TNBC where recent profiling studies report expression in approximately 85% of cases [133]. In contrast to several CAR-T targets that remain preclinical, B7-H3 has gained increasing clinical relevance with multiple antibody and cell-based therapeutic strategies advancing into early-phase clinical trials [134]. Notably, a 2025 clinical study (NCT06347068) is evaluating B7-H3-directed cellular immunotherapy in solid tumors, highlighting its translational potential and positioning B7-H3 as one of the most clinically advanced CAR-T targets currently under investigation for TNBC. Table 3 shows the clinical development status of selected CAR-T targets in TNBC [135].

It is important to note that the majority of CAR-T antigen targets investigated in TNBC, including CSPG4 and ICAM-1, remain at the preclinical stage and are supported primarily by in vitro cell line models and selected in vivo studies. Comprehensive validation of tumor specificity using primary TNBC tissue microarrays and single-cell transcriptomic profiling remains limited. This gap is particularly relevant for assessing potential on-target off-tumor toxicity as several proposed targets may exhibit inducible or low-level expression in normal tissues under inflammatory conditions [136]. Accordingly, rigorous antigen validation across malignant and normal cellular tissues represents a critical prerequisite for safe clinical translation of CAR-T strategies in TNBC.

##### Clinical Evaluation of Cell Therapy in Triple Negative Breast Cancer

Despite being in early stages of development for solid tumors, CAR-T therapy has garnered increasing interest in TNBC due to its capacity for highly specific antigen targeting and potent cytotoxic activity [137]. Several early-phase clinical trials are investigating CAR-T constructs directed against TNBC-associated antigens including MUC1 and mesothelin (NCT02587689 and NCT02580747). These studies aim to evaluate feasibility, safety, and preliminary antitumor activity in patients with advanced TNBC [58,138,139]. Although clinical data remain limited, the initiation of multiple phase I/II trials highlights the growing therapeutic potential of CAR-T approaches in this aggressive breast cancer subtype [140].

##### Limitations of CAR-T Therapy

Although CAR-T therapy has shown remarkable and durable responses in hematologic malignancies [117], its clinical application in solid tumors such as TNBC remains significantly limited by both biological and physical barriers.

Biological limitations include antigen heterogeneity and antigen escape, whereby TNBC tumor cells downregulate or lose expression of the targeted antigen, allowing them to evade CAR-T recognition [141]. In addition, off-tumor toxicity poses a major safety concern as many TNBC-associated antigens are expressed at low levels in normal tissues, potentially leading to unintended tissue damage [114]. Furthermore, the immunosuppressive TME enriched with regulatory T cells, MDSCs, inhibitory cytokines, and checkpoint ligands can impair CAR-T cell expansion, persistence, and cytotoxic activity [57,130,138,142].

Physical limitations are primarily related to CAR-T cells trafficking and infiltration into solid tumor sites. Dense extracellular matrix components, abnormal tumor vasculature, and elevated interstitial pressure collectively restrict CAR-T cell homing and penetration into TNBC tumors, thereby limiting therapeutic efficacy [141]. Altogether, these biological and physical limitations highlight the need for next-generation CAR designs and combination strategies to enhance CAR-T cell performance in TNBC.

#### 3.2.2. Tumor Infiltrating Lymphocyte Therapy

TIL therapy is an ACT approach that utilizes naturally occurring antitumor lymphocytes harvested from the patient tumor [143]. This strategy has gained increasing interest due to its ability to induce durable clinical responses, including complete remissions, particularly in cancers that are refractory to conventional treatments. The therapeutic process involves three major steps, (1) isolating TILs from freshly resected tumor tissue, (2) expanding these lymphocytes ex vivo under good manufacturing practice (GMP) conditions, and (3) reinfusing the expanded autologous TILs into the patient following lymphodepleting conditioning therapy [144].

TNBC is a particularly promising candidate for TIL-based therapy due to its highly immunogenic TME [138,139]. Among all breast cancer subtypes, TNBC exhibits the highest levels of cytotoxic CD8^+^ T-cell infiltration, a feature strongly associated with enhanced immune cytolytic activity and improved clinical outcomes [145]. Large-scale transcriptomic analyses confirm that elevated CD8^+^ T-cell counts correlate with enriched interferon-stimulated gene expression, increased antitumor immune cell infiltration, and significantly better survival in TNBC [146]. Moreover, TNBC tumors with concomitant increases in CD8^+^ and CD4^+^ memory T cells demonstrate even greater survival benefit [147]. These findings not only highlight the intrinsic immunogenicity of TNBC but also support the rationale for leveraging TILs as a therapeutic modality. Additionally, the strong correlation between the CD8^+^ T-cell abundance and the expression of immune checkpoint molecules suggests that TIL-rich tumors may be particularly responsive to combined ACT and checkpoint inhibition strategies.

### 3.3. Oncolytic Virus Platforms

OVs are naturally occurring or genetically engineered viruses that selectively infect, replicate within, and lyse cancer cells while sparing normal tissues [148]. Beyond direct tumor cell destruction (oncolysis), OVs can be utilized for immunotherapy as they stimulate antitumor immunity, releasing TAAs, and activating innate and adaptive immune pathways [149].

OVs used in cancer therapy fall broadly into DNA and RNA virus categories. DNA viruses such as adenovirus, vaccinia virus (VV), and herpes simplex virus-1 (HSV-1) possess large and genetically stable genomes that allow insertion of therapeutic transgenes, including cytokines and costimulatory molecules [149]. This makes them well-suited for engineered OVs aimed at enhancing antitumor immunity in breast cancer and TNBC. RNA viruses including reovirus (RV), vesicular stomatitis virus (VSV), measles virus (MV), and Maraba virus have smaller genomes and rapid replication kinetics, which enable potent direct oncolysis and strong innate immune activation, although they permit more limited genetic manipulation [138].

**Figure 4:**
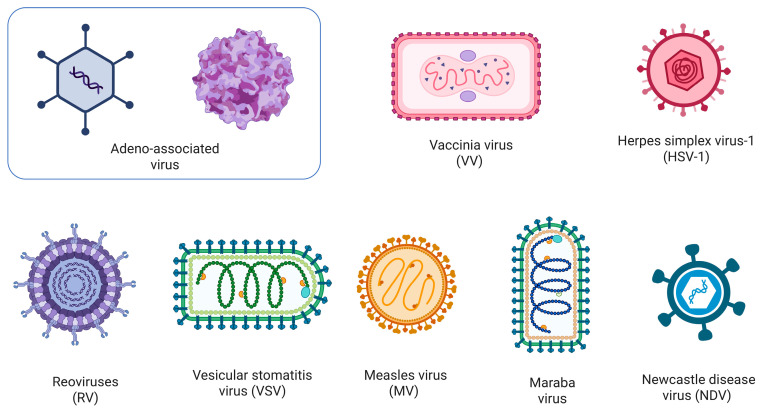
Representative oncolytic virus platforms investigated in TNBC. This figure illustrates key OVs families that have been evaluated in preclinical and early clinical studies for TNBC. DNA-based OVs including adenovirus, vaccinia virus (VV), and HSV-1, possess large stable genomes enabling insertion of immunostimulatory transgenes and are widely used in engineered virotherapy. RNA-based platforms include reoviruses (RV), which naturally replicate in Ras-activated tumor cells, vesicular stomatitis virus (VSV), and Maraba virus, both known for strong immunogenicity and potent oncolytic activity, measles virus (MV), which preferentially infects tumor cells expressing CD46, and Newcastle disease virus (NDV), a nonpathogenic avian paramyxovirus with selective tumor tropism [157]. These diverse viral systems collectively demonstrate the range of OV platforms currently explored for TNBC viroimmunotherapy [148,153,158,159,160]. Created using BioRender.com.

Both DNA- and RNA-based OVs have demonstrated tumor-selective replication, induction of immunogenic cell death, and activation of tumor-specific immune responses in preclinical breast cancer and TNBC models [150,151,152]. Among DNA-based OVs, VV, HSV-1, and oncolytic adenoviruses are the most frequently investigated platforms in TNBC [150,153]. Promisingly, single-stranded positive-sense RNA viruses such as Coxsackievirus and poliovirus have demonstrated rapid oncolytic activity in breast cancer models [154]. Additionally, several single-stranded negative-sense RNA viruses, including VSV [151], MV [155], Maraba [156] and Newcastle disease virus [156], have demonstrated promising preclinical activity in TNBC largely due to their strong immunogenicity and ability to induce immunogenic tumor cell death. Collectively, these viral approaches provide diverse and complementary approaches for exploiting the susceptibility of TNBC to oncolytic viroimmunotherapy. Figure 4 summarizes OVs platforms investigated in TNBC.

#### 3.3.1. Mechanisms of Action of Oncolytic Viruses in Triple Negative Breast Cancer

OVs exert antitumor activity through a combination of direct tumor cell killing and immune system activation. Although the precise mechanisms vary among viral platforms, several conserved processes explain how OVs eliminate tumor cells and stimulate systemic antitumor immunity.

##### Tumor Tropism and Selective Replication

A fundamental requirement for OV efficacy is the ability to selectively enter and replicate within tumor cells. Viral tropism is determined by multiple factors, including expression of viral entry receptors on tumor cells, efficient internalization, and permissiveness to viral protein synthesis [149]. Cancer-specific defects such as impaired antiviral signaling, dysregulated cell-cycle pathways and increased metabolic stress further enhance viral replication [161]. These tumor-specific defects include impaired activation of the protein kinase R (PKR)-eukaryotic initiation factor 2α (eIF2α) (PKR–eIF2α) antiviral pathway, which normally restricts viral protein synthesis in healthy cells but is frequently dysfunctional in cancer cells, thereby permitting selective OV replication [161]. In TNBC, engineered VV has shown improved tropism by incorporating tumor-targeting elements such as single-chain antibodies against highly expressed factors (e.g., vascular endothelial growth factor (VEGF)) [151]. Additional strategies to enhance tumor selectivity include targeting TNBC-associated surface antigens such as B7-H3 (CD276) and nectin-4 [134,162].

##### Direct Oncolysis

Following successful infection, OVs replicate within tumor cells and induce cell death through multiple lytic pathways, including apoptosis, necrosis, and autophagy [163]. In addition to these pathways, OV infection can induce inflammatory forms of cell death such as pyroptosis through activation of caspase-1-dependent gasdermin signaling, contributing to immunogenic tumor cell destruction [164]. Viral replication can trigger endoplasmic reticulum stress and activate the unfolded protein response, ultimately leading to tumor-cell death [165]. Sustained viral replication also imposes profound metabolic stress on tumor cells, leading to ATP depletion, disruption of biosynthetic pathways, and metabolic exhaustion that further promotes cell death [151]. Genetic modifications can enhance this effect. For example, insertion of the pro-apoptotic TNF-related apoptosis-inducing ligand (TRAIL) gene into oncolytic adenovirus significantly inhibited TNBC growth and metastasis through death receptor 4/death receptor 5 (DR4/DR5)-mediated apoptotic signaling [166].

##### Activation of Innate Immunity

OV infection stimulates strong innate immune responses within the TME. DCs, macrophages, and natural killer (NK) cells are recruited to infected tumor sites in response to pathogen-associated and damage-associated molecular patterns (PAMPs and DAMPs) [167]. Engagement of pattern-recognition receptors such as Toll-like receptors induces DC maturation and IL-6 and -12, and Tumor necrosis factor alpha (TNF-α) cytokine release, enhancing local inflammation and supporting antitumor activity [151,168,169].

##### Induction of Adaptive Antitumor Immunity

**Figure 5:**
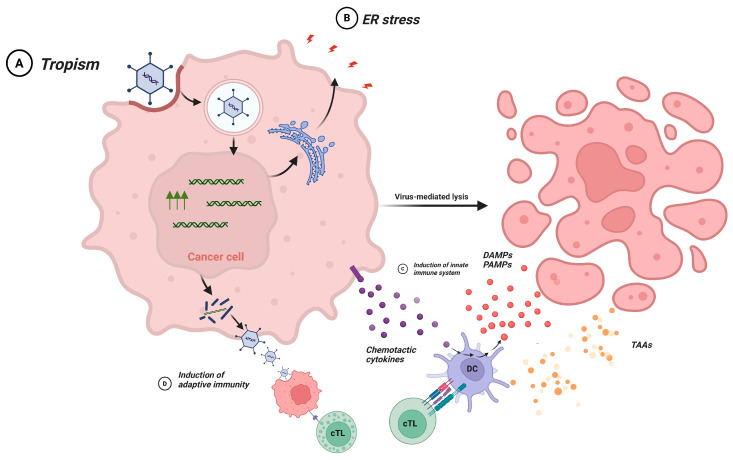
Mechanisms of antitumor activity of oncolytic viruses. (**A**) OVs selectively enter cancer cells through receptor-mediated attachment and internalization followed by efficient intracellular replication. (**B**) Viral replication induces endoplasmic reticulum (ER) stress and cellular dysfunction, leading to immunogenic forms of cell death. (**C**) Virus-mediated lysis releases DAMPs and PAMPs, which activate DCs and trigger production of pro-inflammatory and chemotactic cytokines. (**D**) DCs present TAAs and neoantigens released after oncolysis to cytotoxic T lymphocytes (cTLs), enhancing tumor-specific adaptive immune responses [150,151,172]. Created using BioRender.com.

Oncolysis leads to the release of TAAs and neoantigens, which are taken up and cross-presented by DCs to CD4^+^ and CD8^+^ T cells via MHC-II and MHC-I pathways, respectively [170]. This promotes clonal expansion of tumor-specific cytotoxic T cells that traffic back to the tumor site. OVs therefore function as in situ cancer vaccines, amplifying systemic antitumor immunity. Strategies to further potentiate this effect include engineering OVs to express cytokines or costimulatory molecules that enhance T-cell activation [171]. Collectively, these mechanisms, tumor-selective replication, direct oncolysis, innate immune activation, and the potent stimulation of adaptive immunity form the basis of OV-mediated immunotherapy and support the rationale for their investigation in TNBC (Figure 5).

#### 3.3.2. Oncolytic Viruses in Triple Negative Breast Cancer

Oncolytic virotherapy offers a promising therapeutic approach for TNBC [160]. Multiple preclinical studies have demonstrated that OVs can selectively infect TNBC cells, induce tumor-specific cell death, and stimulate robust antitumor immunity [149]. Several engineered viruses, particularly those incorporating immunomodulatory cytokines, have produced enhanced therapeutic activity in TNBC models. Table 4 summarizes representative OVs evaluated in TNBC research.

To enhance the therapeutic potency of OVs, many studies have engineered viral vectors to express immunoregulatory or pro-apoptotic cytokines [176]. One of the most widely studied cytokines is IL-24, which has direct tumor-suppressive activity including inhibition of proliferation and induction of apoptosis [176]. In TNBC models, an adenovirus engineered to express IL-24 (CNHK600-IL24) demonstrated significantly increased antitumor activity and improved survival in both nude-mouse TNBC xenografts and metastatic breast cancer models, confirming the potential of adenoviral OVs in TNBC therapy [172].

Similarly, an HSV-1-based OV engineered to express IL-12 (G47Δ-mIL12) showed potent cytotoxicity against TNBC cells in vitro and markedly suppressed tumor growth and metastasis in vivo in the 4T1 syngeneic model [173]. These results highlight the value of cytokine-armed OVs in stimulating antitumor immunity in TNBC. Furthermore, recombinant VV expressing IL-24 (VG9-IL-24) demonstrated selective cytotoxicity against TNBC cells without harming normal cells [177]. In MDA-MB-231 xenograft models, VG9-IL-24 significantly slowed tumor progression, prolonged survival, and increased OS rates [174,177]. Since MDA-MB-231 is a TNBC cell line [178], the outcome of this investigation presented clear evidence for potential use of VV in TNBC therapy.

Finally, vesicular stomatitis virus (VSVd51), an engineered RNA-based OV with enhanced tumor selectivity, demonstrated potent cytotoxicity in both murine and human TNBC cells [179]. Importantly, VSVd51 also recruited NK and CD8^+^ T cells into the TME, suggesting potential synergy with ICIs and supporting combination OV-ICI strategies for TNBC [175].

An important clinically validated example of cytokine-armed oncolytic virotherapy is talimogene laherparepvec (T-VEC), a granulocyte-macrophage colony-stimulating factor (GM-CSF) expressing HSV-1 that is FDA-approved for the treatment of advanced melanoma [180]. Although not approved for breast cancer, T-VEC has demonstrated the ability to induce immunogenic tumor cell death, enhance dendritic cell recruitment, and promote systemic antitumor immune responses [181]. Preclinical studies and early translational investigations suggest that GM-CSF-armed HSV-1 platforms may similarly enhance antitumor immunity in TNBC, supporting their evaluation as combination partners with ICIs [180].

Collectively, these preclinical findings highlight the strong therapeutic potential of OVs in TNBC and support continued development of engineered viral platforms alone or in combination with other immunotherapies to overcome the immunosuppressive TNBC microenvironment. It should be acknowledged that much of the preclinical evidence supporting oncolytic virotherapy in TNBC is derived from xenograft and syngeneic murine models. These models differ substantially from human tumors with respect to antiviral innate immune responses, particularly type I interferon signaling, which can strongly restrict viral replication in immunocompetent hosts. Species-specific differences in interferon responsiveness and immune regulation may therefore attenuate OV activity in human tumors compared with preclinical models. Consequently, while these studies provide important mechanistic and proof-of-concept insights, careful interpretation and clinical validation are required before extrapolating TNBC-specific efficacy to human settings.

### 3.4. Monoclonal Antibodies

mAbs are laboratory-engineered immunoglobulins designed to recognize and bind with high specificity to antigens expressed on cancer cells [182]. Their therapeutic activity is mediated through several mechanisms, including blockade of oncogenic signaling, recruitment of immune effector mechanisms, and targeted delivery of cytotoxic payloads when formulated as antibody-drug conjugates (ADCs) [183]. Among these strategies, ADCs have become particularly important in TNBC where few targeted therapies exist. Sacituzumab govitecan-hziy (Trodelvy), the first ADC approved specifically for mTNBC, received accelerated FDA approval on 22 April 2020 for patients who had previously received at least two lines of therapy [184]. This approval was supported by data from the IMMU-132-01 phase I/II multicohort trial (NCT01631552), which demonstrated meaningful objective responses and clinical benefit in heavily pretreated TNBC patients [185]. More recently, results from the ASCENT-03 (NCT05382299) and ASCENT-04 trials (NCT05382286) have confirmed the efficacy of sacituzumab govitecan as a first-line treatment option in patients with mTNBC who are ineligible for immunotherapy, expanding its role beyond the late-line setting. Trastuzumab deruxtecan (T-DXd; Enhertu), an ADC initially developed for HER2-positive breast cancer, has demonstrated significant clinical activity in tumors with low HER2 expression, defined as IHC score 1+ or 2+ with negative in situ hybridization (ISH−) [186]. This expanded indication includes a subset of TNBC classified as HER2-low, highlighting a new therapeutically actionable category within TNBC.

#### Mechanism of Action of Sacituzumab Govitecan (Trodelvy)

Sacituzumab govitecan is an ADC composed of a humanized mAB targeting trophoblast cell-surface antigen 2 (Trop-2) conjugated to SN-38, the active metabolite of irinotecan, through a moderately stable pH-sensitive linker [187]. Trop-2 is a transmembrane glycoprotein involved in calcium signaling and tumor cell proliferation and its overexpression correlates with enhanced metastatic potential and poor prognosis in breast cancer [188]. Importantly, Trop-2 is highly expressed in approximately 90% of TNBC tumors, making it a clinically relevant therapeutic target [184,189,190].

Once sacituzumab govitecan binds Trop-2 on TNBC cells, the ADC-receptor complex undergoes internalization and the acidic environment of the lysosome triggers cleavage of the linker, releasing SN-38 intracellularly [191]. SN-38 inhibits topoisomerase I by stabilizing the cleavable complex between the enzyme and DNA, thereby preventing re-ligation and ultimately inducing S-phase-specific DNA damage and apoptosis [192]. The high drug-to-antibody ratio (DAR) ~7.6 ensures delivery of therapeutically effective SN-38 concentrations, even in cells with moderate Trop-2 expression [190,193].

**Figure 6:**
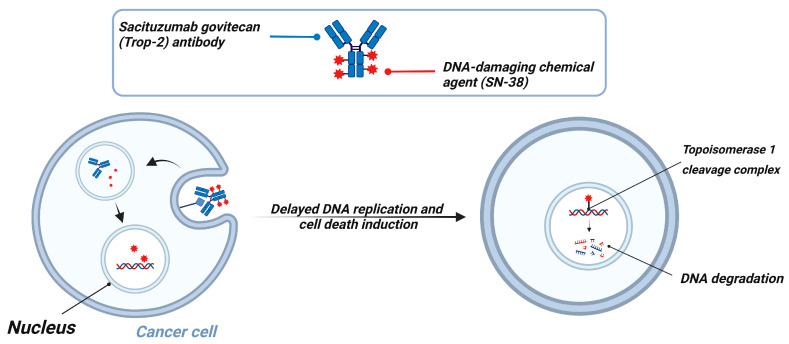
Mechanism of action of sacituzumab govitecan (Trodelvy) in TNBC. Sacituzumab govitecan is an ADC composed of an anti-Trop-2 mAB linked to the cytotoxic topoisomerase-I inhibitor SN-38. After binding to Trop-2, which is highly expressed on TNBC cells, the ADC is internalized and releases SN-38 intracellularly. SN-38 binds to and stabilizes the topoisomerase I-DNA cleavage complex, preventing re-ligation of single-strand breaks, delaying DNA replication, and inducing S-phase-specific cell death. This figure illustrates antibody binding, internalization, SN-38 release, and subsequent DNA degradation leading to apoptotic cell death [196]. Created with BioRender.com.

Compared with irinotecan, sacituzumab govitecan is associated with a lower incidence of severe diarrhea, likely because SN-38 is delivered in its active non-glucuronidated form directly inside tumor cells rather than systemically [194]. However, treatment-related toxicities remain clinically significant with neutropenia reported in approximately 46% of patients and grade ≥ 3 diarrhea occurring in ~11% of cases [195]. Preclinical studies further demonstrated that sacituzumab govitecan induces strong apoptotic signals such as caspase-3 activation, Poly(ADP-ribose) polymerase (PARP) cleavage, and upregulation of p21^ wild-type p53-activated fragment 1 (WAF1)/Cip1 while exhibiting superior antitumor efficacy relative to irinotecan in xenograft TNBC models (Figure 6) [193].

## 4. Mechanisms of Resistance to Immunotherapy in Triple Negative Breast Cancer

Despite being an immunologically active breast cancer subtype characterized by relatively high TILs, TNBC frequently develops resistance to immunotherapy [197]. This resistance arises from a combination of tumor-intrinsic features and an immunosuppressive TME that collectively limit effective T-cell activation, antigen presentation, and immune-mediated cytotoxicity [198,199].

### 4.1. Tumor-Intrinsic Mechanisms

Several genomic and metabolic adaptations support immune escape in TNBC. Loss of MMR function increases TMB and may enhance responsiveness to ICIs [200], but MMR-proficient TNBC tends to be intrinsically resistant as intact MMR permits error-free DNA repair and reduces neoantigen formation [201]. Upregulation of DNA repair proteins such as O6-methylguanine-DNA methyltransferase (MGMT), X-ray repair cross-complementing protein 1 (XRCC1), apurinic/apyrimidinic endonuclease 1 (APE1), flap endonuclease 1 (FEN1), excision repair cross-complementation group 1 (ERCC1), and long non-coding RNA in non-homologous end joining pathway 1 (LINP1) strengthens the repair of DNA damage induced by cytotoxic immune mechanisms, allowing tumor cells to survive immune-mediated DNA stress [202]. Recent evidence suggests that increased expression of DNA repair scaffold proteins, particularly that of XRCC1, may represent an emerging biomarker of resistance to PD-1 blockade, including that of pembrolizumab by limiting immune-mediated DNA damage and reducing tumor immunogenicity [198]. It should be acknowledged that evidence linking DNA repair protein upregulation (including MGMT, XRCC1, and related scaffold or repair factors) to immunotherapy resistance in TNBC is derived from heterogeneous datasets and remains largely associative. Emerging studies suggest that enhanced DNA repair capacity may limit immune-mediated DNA damage and antigenicity. However, definitive causal validation remains limited, particularly across molecular TNBC subtypes such as BL1A and BLIS, using subtype-resolved transcriptomic analyses or functional clustered regularly interspaced short palindromic repeats (CRISPR)-based screening. Consequently, these DNA repair pathways should be viewed as candidate contributors to immune resistance that warrant further mechanistic and subtype-specific investigation rather than as fully established determinants of ICI resistance.

Metabolic reprogramming also plays a major role in immune resistance. TNBC cells exhibit enhanced glycolysis (Warburg effect), fatty-acid oxidation (FAO), and oxidative phosphorylation, creating a nutrient-depleted acidic microenvironment [203]. High lactate output and hypoxia impair T-cell effector function and favor immune evasion [204]. Notably, enhanced lipid metabolism and FAO have recently been proposed as metabolic biomarkers of ICI resistance, including reduced responsiveness to pembrolizumab by promoting T-cell exhaustion and supporting tumor cell survival under immune pressure [205].

### 4.2. Anti-Apoptotic and Survival Pathways

TNBC frequently overexpresses BCL-2 family members (e.g., BCL-2 and MCL-1), reducing susceptibility to cytotoxic T-cell and NK-cell-mediated apoptosis [206]. Autophagy activation further enables TNBC cells to resist immune-mediated stress by recycling damaged organelles and maintaining metabolic activity [207].

### 4.3. TME-Mediated Resistance

The TME is a central driver of immune evasion and immunotherapy resistance in TNBC through coordinated cellular and molecular interactions that suppress antitumor immunity. Cancer-associated fibroblasts (CAFs) contribute to immune exclusion by secreting the chemokine CXCL12, which promotes the accumulation of immunosuppressive regulatory T cells (Tregs) while preventing effective infiltration of cytotoxic T lymphocytes into the tumor core [208]. In parallel, tumor-associated macrophages (TAMs), frequently polarized toward an anti-inflammatory M2 phenotype by TNBC-derived granulocyte colony-stimulating factor (G-CSF), suppress T-cell effector function and support cancer stem-like properties that favor tumor persistence [17]. These immunosuppressive effects are further reinforced through immune checkpoint and innate immune evasion pathways. Both TAMs and TNBC cells can express PD-L1, leading to direct inhibition of cytotoxic T-cell activation via PD-1 engagement [209]. Additionally, TNBC cells frequently overexpress CD24, which interacts with Siglec-10 on macrophages to deliver a “don’t-eat-me” signal, thereby limiting phagocytic clearance and promoting immune escape [210]. Hypoxic conditions within the TME amplify these resistance mechanisms by enhancing PD-L1 expression and reinforcing immune suppression [211]. Moreover, extracellular vesicles (EVs) released by TNBC cells disseminate immunosuppressive proteins and microRNAs throughout the TME, enabling both local and systemic propagation of resistance signals [212].

Overall, immunotherapy resistance in TNBC arises from the interplay of intrinsic tumor cell survival pathways (DNA repair, metabolism, and anti-apoptotic signaling) and an immunosuppressive microenvironment that restricts effective antigen presentation, T-cell infiltration, and cytotoxic function. These resistance mechanisms highlight the need for rational combination strategies such as ICIs with OVs, PARP inhibitors (PARPi), or metabolic modulators to enhance immunotherapy responsiveness in TNBC.

## 5. Strategies to Improve Immunotherapy in Triple Negative Breast Cancer

Combination strategies have emerged as a major approach to enhance the clinical benefit of immunotherapy in TNBC. Because TNBC is heterogeneous and characterized by multiple mechanisms of immune escape, combining ICIs with chemotherapy, targeted therapies, or additional immunotherapies has shown improved responses compared with monotherapy in several clinical trials [213].

### 5.1. Anti-PD-1/PD-L1 Inhibitors Combined with Chemotherapy

Chemotherapy induces several immunomodulatory changes within the TME that enhance the efficacy of ICIs. These include increased tumor antigen release, upregulation of MHC-I, stimulation of dendritic cell activation, and priming of CD8^+^ T-cell responses [214]. Furthermore, chemotherapy can induce immunogenic cell death, thereby increasing tumor visibility to the immune system [215]. A preclinical study showed that paclitaxel enhances DC antigen presentation and CD8^+^ T-cell priming when combined with PD-1 blockade in TNBC models [216]. Clinical trials have demonstrated that PD-1/PD-L1 inhibitors combined with chemotherapy outperform single-agent ICIs in mTNBC [217]. Specifically, addition of pembrolizumab to chemotherapy significantly improves treatment efficacy as demonstrated in the KEYNOTE-522 trial (NCT03036488), which reported a pCR rate of approximately 64.8% [218].

A meta-analysis by Han et al. found that adding ICIs to chemotherapy improves TNBC outcomes [11]. In neoadjuvant therapy, it significantly increased pCR and event-free survival (EFS), including in both PD-L1-positive and PD-L1-negative patients. In the adjuvant setting, it prolonged PFS with greater benefit in PD-L1-positive tumors. However, the combination increased the incidence of overall and ≥3 grade adverse events, particularly immune-related thyroid toxicity, highlighting the need for careful safety monitoring [218].

The IMpassion130 phase III trial (NCT02425891) evaluated atezolizumab plus nab-paclitaxel versus placebo plus nab-paclitaxel in advanced TNBC. In the intention-to-treat (ITT) population, combination therapy improved median PFS by 1.7 months compared with control groups with a media PFS improvement of 2.5 months in the PD-L1-positive subgroup [219,220]. In contrast, the IMpassion131 trial (NCT03125902), which evaluated atezolizumab plus paclitaxel, did not demonstrate PFS or OS improvement compared to paclitaxel alone. This lack of benefit may reflect pharmacokinetic and immunologic differences between solvent-based paclitaxel and nab-paclitaxel, including lower intra-tumoral drug delivery and reduced chemotherapy-induced immune priming. These findings prompted an FDA advisory stating that paclitaxel should not replace nab-paclitaxel when combined with atezolizumab for mTNBC (mTNBC) [221]. Subsequently, the accelerated FDA approval of atezolizumab plus nab-paclitaxel for PD-L1-positive mTNBC was voluntarily withdrawn in 2021 and this regimen is no longer routinely recommended in current practice, although it remains approved in some regions, such as the EU [219].

The phase III KEYNOTE-355 trial (NCT02819518) in patients with previously untreated metastatic or unresectable locally recurrent TNBC showed that pembrolizumab plus chemotherapy significantly improved PFS and OS compared with chemotherapy alone in the PD-L1–positive subgroup (CPS ≥ 10) [222].

Evidence also suggests that select TNBC subgroups may derive meaningful benefit from chemotherapy-free immune-checkpoint priming, particularly when immune activation is robust [223]. In the phase 2 adaptive BELLINI trial (NCT03815890), Nederlof et al. reported neoadjuvant nivolumab plus ipilimumab without chemotherapy achieving immune activation in over half of treated patients with major pathological response observed in approximately 53% of participants [224]. These data support ongoing interest in biomarker-driven de-escalation strategies for carefully selected TNBC patients while emphasizing that such approaches remain investigative and not yet a universal clinical standard [224].

Collectively, these findings support the use of PD-1/PD-L1 inhibitors with chemotherapy as a more effective strategy than monotherapy for TNBC.

### 5.2. Anti-PD-1/PD-L1 Combined with Targeted Therapy

Combining immunotherapy with targeted agents offers a compelling approach to overcoming resistance in TNBC [225].

#### 5.2.1. PARP and Immune Checkpoint Inhibitors

PARPi induce synthetic lethality in BRCA1/2-mutated TNBC and may enhance antitumor immunity by increasing neoantigen generation, promoting tumor cell lysis, antigen release, and upregulation of PD-L1 expression [226,227]. The TOPACIO/KEYNOTE-162 trial (NCT02657889) evaluated pembrolizumab in combination with niraparib in patients with pretreated advanced or mTNBC. In 47 evaluable patients, the ORR was 21% and the disease control rate was 49%, higher than expected with PD-1 monotherapy in similar populations [228]. Several additional trials are evaluating PARPi–ICI combinations including durvalumab plus olaparib (DORA/NCT03167619, NCT03801369) and atezolizumab plus olaparib (NCT02849496). Another study combining rucaparib with atezolizumab in platinum-sensitive or homologous-recombination-deficient TNBC reported tolerable safety and preliminary antitumor activity, supporting the feasibility of PARPi and ICI combinations in TNBC [226,227,229,230].

#### 5.2.2. MAPK Pathway and Immune Checkpoint Inhibitors

The mitogen-activated protein kinase (MAPK) pathway, frequently activated in TNBC, contributes to tumor proliferation and apoptosis resistance [231]. Mechanistically, mitogen-activated protein kinase (MEK) inhibition has been shown to enhance antitumor immune responses by increasing type I and II interferon signaling, upregulating MHC-I expression, and promoting cytokine programs that improve tumor antigen presentation and T-cell recognition [232]. These immunomodulatory effects provide a strong biological rationale for combining MEK inhibitors with ICB [233,234]. The COLET trial (NCT02322814) evaluated the MEK1/2 inhibitor cobimetinib in combination with atezolizumab plus paclitaxel/nab-paclitaxel as a first-line therapy in advanced TNBC. Interim results showed ORRs of 34% (paclitaxel arm) and 29% (nab-paclitaxel arm), indicating potential benefit from MEK inhibition combined with immunotherapy. Importantly, because sustained MEK inhibition may impair T-cell proliferation and effector function, emerging strategies favor pulsatile or sequential dosing schedules to maximize tumor immunogenicity while minimizing T-cell suppression [235]. Beyond PARP and MAPK inhibition, other targeted pathways (e.g., phosphoinositide 3-kinase (PI3K)/Protein kinase B (AKT)/mammalian target of rapamycin (mTOR), janus kinase (JAK)/signal transducer and activator of transcription (STAT), epigenetic regulators) and ICI combinations are being actively studied [236].

### 5.3. Anti-PD-1/PD-L1 Inhibitors Combined with Other Immunotherapies

TNBCs frequently express high levels of additional immune checkpoints such as CTLA-4, LAG-3, and TIGIT that may contribute to resistance to the PD-(L)1 blockade [213,237]. PD-1/PD-L1 inhibition further increases the expression of these suppressive receptors, providing rationale for dual-checkpoint combinations [238]. A small single-arm trial (NCT02536794) combining durvalumab plus tremelimumab (CTLA-4 inhibitor) in mTNBC demonstrated an ORR of 43% in three out of seven patients with an acceptable safety profile. In addition, a phase Ib/II study (NCT03872791) evaluating KN046, a bispecific antibody targeting PD-L1 and CTLA-4, combined with nab-paclitaxel in advanced TNBC reported encouraging preliminary activity, supporting further development of dual-checkpoint immunotherapy strategies [239]. A recent systematic review confirmed that CTLA-4/PD-1 or CTLA-4/PD-L1 co-blockade increases the presence of immune activation markers and response rates specifically in TNBC [240].

Beyond classical checkpoint-based combinations, emerging evidence highlights that reversing transcriptional programs linked to immune suppression can enhance ICI efficacy in TNBC. Famta et al. demonstrated that inflammatory signaling from CAFs drives EMT-linked immune escape in part via PD-1/PD-L1 upregulation [241]. Pharmacologic blockade and cytokine reprogramming strategies reduced CAF-derived mediators including the IL-6/IL-8/chemokine axis, decreased EMT activity, and partially restored T-cell responsiveness, supporting CAF-focused immune reactivation as a complementary approach to improve immunotherapy durability [208]. A pan-cancer stromal-immunity meta-analysis further validated that CAF reprogramming improves T-cell infiltration and enhances durability of ICI responses in TNBC-enriched cohorts [242]. However, recent clinical consensus emphasizes that these high-response combination strategies are associated with increased immune-related toxicity, underscoring the need for careful patient selection, optimized dosing schedules, and vigilant safety monitoring when deploying dual or multi-agent immunotherapy regimens [243].

## 6. Conclusions

Immunotherapy has introduced significant progress in the management of TNBC, a subtype historically defined by limited therapeutic options and poor outcomes. Incorporation of ICIs into neoadjuvant, adjuvant, and metastatic treatment settings, particularly when combined with immunogenic chemotherapy, has demonstrated meaningful clinical benefit and has reshaped the therapeutic landscape [244]. Furthermore, combinations with targeted agents such as PARPi, MEK inhibitors, and oncolytic virotherapies have opened new avenues for patients with refractory or previously unresponsive TNBC.

However, the heterogeneity of TNBC and the complexity of its immune microenvironment continue to limit the efficacy of immunotherapy in a substantial proportion of patients. Accumulating evidence indicates that treatment response is closely linked to specific tumor and immune features including TIL density, mismatch repair status, TMB, PD-L1 expression, and broader immune checkpoint profiles. Consequently, optimizing patient selection through robust clinically validated biomarkers and potentially through molecular subtyping of the immune microenvironment will be essential to improve therapeutic precision and maximize benefit.

As our understanding of TNBC immunobiology deepens, rational combination strategies and biomarker-guided approaches hold the promise of overcoming current resistance mechanisms and further expanding the therapeutic potential of immunotherapy. Continued translational and clinical research will be crucial to refine these strategies and ultimately improve outcomes for patients with this aggressive disease.

Importantly, emerging therapeutic strategies increasingly aim to convert immunologically “cold” TNBC tumors into “hot” immune-responsive states [197]. Targeted agents such as PARPi, MEK inhibitors, and ADCs not only exert direct antitumor effects but also enhance tumor immunogenicity, antigen presentation, and immune infiltration, thereby sensitizing tumors to immune checkpoint blockade.

Looking forward, the next decade of TNBC immunotherapy is likely to focus on integrating spatial and temporal biomarkers to optimize treatment selection and sequencing. Approaches that capture intra-tumoral heterogeneity, immune-tumor spatial interactions, and dynamic changes during disease evolution will be essential to deliver the right therapy at the optimal stage, ultimately improving the durability and precision of immunotherapy responses in TNBC.

## Figures and Tables

**Table 1:** A descriptive summary of selected clinical trials evaluating anti-PD-1 and anti-PD-L1 monotherapy in TNBC.

Trial	Regimen	Status/Key Results	Ref.
KEYNOTE-012 (Phase Ib); NCT01848834	Pembrolizumab	Completed; ORR: 18.5% in PD-L1-positive mTNBC	[67]
KEYNOTE-086A (Phase II); NCT02954874	Pembrolizumab	Completed; ORR: 5.3% in previously treated, unselected mTNBC	[61,62]
KEYNOTE-086B (Phase II), first-line; NCT02447003	Pembrolizumab	Completed; ORR: 21.4% in treatment-naïve, PD-L1-positive mTNBC	[61]
KEYNOTE-119 (Phase III); NCT02555657	Pembrolizumab vs. chemotherapy	Completed; no significant improvement in PFS or OS	[52,63]
JAVELIN (Phase I); NCT01772004	Avelumab	Completed; ORR: 5.2%; responses higher in PD-L1-positive vs. PD-L1-negative tumors (22.2% vs. 2.6%); monotherapy remains modest	[66]
Atezolizumab Multi-cohort (Phase I) (PCD4989g trial; NCT01375842)	Atezolizumab	Completed; first-line ORR: 24%; later-line ORR: 6%; higher responses in PD-L1-positive tumors	[40]

CPS, combined positive score; mTNBC, metastatic triple-negative breast cancer; ORR, objective response rate; OS, overall survival; PD-L1, programmed death-ligand 1; PFS, progression-free survival.

**Table 2:** Immune-related adverse events associated with ICIs.

Organ/System Affected	Immune-Related Adverse Events (irAEs)	Ref.
Cardiovascular	Myocarditis; Pericarditis	[108]
Endocrine system	Diabetes mellitus; Primary hypothyroidism; Hyperthyroidism; Adrenal insufficiency	
Gastrointestinal	Colitis; Hepatitis	
Haematological	Aplastic anemia; Acquired hemophilia; Hemolytic uremic syndrome	
Lung	Pneumonitis	
Nervous system	Meningitis; Encephalitis; Myasthenia gravis; Autonomic neuropathy	
Ocular	Iritis; Uveitis	
Renal	Immune-mediated nephritis	
Skin	Maculopapular rash; Pruritus; Dermatitis; Severe cutaneous reactions	

**Table 3:** Clinical development status of selected CAR-T cell targets in TNBC.

Target Antigen	Therapeutic Strategy	Clinical Status (2025)	Representative Trials
B7-H3 (CD276)	Autologous CAR-T cells targeting B7-H3 in TNBC	Phase I	NCT06347068
Mesothelin	Treatment of relapsed or chemotherapy-refractory advanced malignancies with CART-meso	Phase I	NCT02580747
MUC1/MUC1-C	Phase I/II study of anti-mucin1 (MUC1) CAR-T cells for patients with MUC1^+^ advanced refractory solid tumor	Phase I/II	NCT02587689
NKG2D ligands	Allogeneic NKG2DL-targeting CAR-γδ T cells (CTM-N2D) in advanced cancers (ANGELICA)	Phase I	NCT05302037

CART-meso, mesothelin-targeted chimeric antigen receptor T cells; MUC1-C, mucin 1 C-terminal subunit; NKG2D, natural killer group 2, member D; TNBC, triple-negative breast cancer.

**Table 4:** Oncolytic viruses investigated in TNBC preclinical models.

Virus	Gene Inserted/Modification	Mechanism	Ref.
Adenovirus (dsDNA)	CNHK600-IL24	IL-24-armed adenovirus inducing apoptosis in TNBC cells	[172]
Herpes simplex virus (dsDNA)	G47Δ-mIL12	IL-12-expressing HSV enhancing antitumor immunity and reducing tumor growth/metastasis	[173]
Vaccinia virus (dsDNA)	VG9-IL-24	IL-24-armed vaccinia virus inducing apoptosis in TNBC cells	[174]
Vesicular stomatitis virus (ss-RNA)	VSVd51	Matrix-protein mutation increasing tumor selectivity and immunogenicity	[175]

CNHK600, chimeric oncolytic herpes simplex virus strain CNHK600; dsDNA, double-stranded DNA; G47Δ (G47), third-generation oncolytic herpes simplex virus; HSV, herpes simplex virus; IL-12, interleukin 12; IL-24, interleukin 24; ssRNA, single-stranded RNA; TNBC, triple-negative breast cancer; VG9, attenuated oncolytic herpes simplex virus strain VG9; VSV, vesicular stomatitis virus; VV, vaccinia virus.

## Data Availability

No new data were created or analyzed in this study.

## Abbreviations

The following abbreviations are used in this manuscript:

4-1BB	Tumor necrosis factor receptor superfamily member 9
ACT	Adoptive cell therapy
ADC	Antibody–drug conjugate
APC	Antigen-presenting cell
AKT	Protein kinase B
APE1	Apurinic/apyrimidinic endodeoxyribonuclease 1
B7-1	Cluster of differentiation 80
B7-2	Cluster of differentiation 86
BiTE	Bispecific T-cell engager
BRCA1/2	Breast cancer susceptibility genes 1 and 2
CAFs	Cancer-associated fibroblasts
CAR	Chimeric antigen receptor
CAR-T	Chimeric antigen receptor T cells
CD3ζ	Cluster of differentiation 3 zeta
CD8^+^	Cluster of differentiation 8–positive T cells
CPS	Combined positive score
CRISPR	Clustered regularly interspaced short palindromic repeats
CSPG4	Chondroitin sulphate proteoglycan 4
CTL	Cytotoxic T lymphocyte
CTLA-4	Cytotoxic T-lymphocyte–associated protein 4
CXCL12	C-X-C motif chemokine ligand 12
DAMPs	Damage-associated molecular patterns
DAR	Drug-to-antibody ratio
DCs	Dendritic cells
DFS	Disease-free survival
DR4	Death receptor 4
DR5	Death receptor 5
EFS	Event-free survival
EGFR	Epidermal growth factor receptor
eIF2α	Eukaryotic initiation factor 2 alpha
EMT	Epithelial–mesenchymal transition
ER	Estrogen receptor
ERCC1	Excision repair cross-complementation group 1
EVs	Extracellular vesicles
FAO	Fatty acid oxidation
FDA	Food and Drug Administration
FEN1	Flap endonuclease 1
FRα	Folate receptor alpha
GEP	Gene expression profile
GM-CSF	Granulocyte-macrophage colony-stimulating factor
G-CSF	Granulocyte colony-stimulating factor
GMP	Good manufacturing practice
HER2	Human epidermal growth factor receptor 2
HSV-1	Herpes simplex virus type 1
ICAM-1	Intercellular adhesion molecule 1
ICI	Immune checkpoint inhibitor
ICIs	Immune checkpoint inhibitors
ICOS	Inducible T-cell co-stimulator
IFN	Interferon
IgG1	Immunoglobulin G1
IgG4	Immunoglobulin G4
IHC	Immunohistochemistry
IL	Interleukin
IrAEs	Immune-related adverse events
ISH	In situ hybridization
ITIM	Immunoreceptor tyrosine-based inhibitory motif
ITT	Intention-to-treat
JAK	Janus kinase
LAG-3	Lymphocyte activation gene 3
LINP1	Long intergenic non-protein coding RNA, p53-induced transcript
mAbs	Monoclonal antibodies
MAPK	Mitogen-activated protein kinase
MDSCs	Myeloid-derived suppressor cells
MEK	Mitogen-activated protein kinase kinase
MGMT	O6-methylguanine-DNA methyltransferase
MHC	Major histocompatibility complex
MHC-I	Major histocompatibility complex class I
MHC-II	Major histocompatibility complex class II
MMR	Mismatch repair
MSI	Microsatellite instability
MSLN	Mesothelin
mTNBC	Metastatic triple-negative breast cancer
mTOR	Mechanistic target of rapamycin
MV	Measles virus
NDV	Newcastle disease virus
NK	Natural killer
NKG2DL	Natural killer group 2D ligands
ORR	Objective response rate
OS	Overall survival
OVs	Oncolytic viruses
OX-40	Tumor necrosis factor receptor superfamily member 4
p21^WAF1/Cip1^	Cyclin-dependent kinase inhibitor 1A
PAMPs	Pathogen-associated molecular patterns
PARP	Poly(ADP-ribose) polymerase
PARPi	Poly(ADP-ribose) polymerase inhibitors
pCR	Pathological complete response
PD-1	Programmed cell death protein 1
PD-L1	Programmed death-ligand 1
PD-L2	Programmed death-ligand 2
PFS	Progression-free survival
PI3K	Phosphoinositide 3-kinase
PKR	Protein kinase R
PR	Progesterone receptor
ROR1	Receptor tyrosine kinase-like orphan receptor 1
RV	Reovirus
SN-38	7-ethyl-10-hydroxycamptothecin
STAT	Signal transducer and activator of transcription
TAAs	Tumor-associated antigens
TAMs	Tumor-associated macrophages
TEM8	Tumor endothelial marker 8
TIGIT	T-cell immunoreceptor with Ig and ITIM domains
TILs	Tumor-infiltrating lymphocytes
TIM-3	T-cell immunoglobulin and mucin-domain containing protein 3
TMB	Tumor mutational burden
TME	Tumor microenvironment
TNBC	Triple-negative breast cancer
TNF-α	Tumor necrosis factor alpha
TRAIL	Tumor necrosis factor–related apoptosis-inducing ligand
Tregs	Regulatory T cells
TROP2	Trophoblast cell-surface antigen 2
TRUCKs	T cells redirected for universal cytokine killing
VEGF	Vascular endothelial growth factor
VISTA	V-domain Ig suppressor of T-cell activation
VSVd51	Vesicular stomatitis virus d51 mutant
VV	Vaccinia virus
XRCC1	X-ray repair cross-complementing protein 1

## References

[1] Siegel R.L., Giaquinto A.N., Jemal A. (2024). Cancer Statistics, 2024. CA Cancer J. Clin..

[2] Xu Y., Gong M., Wang Y., Yang Y., Liu S., Zeng Q. (2023). Global Trends and Forecasts of Breast Cancer Incidence and Deaths. Sci. Data.

[3] Patel K.K., Hassan D., Nair S., Tejovath S., Kahlon S.S., Peddemul A., Sikandar R., Mostafa J.A. (2022). Role of Immunotherapy in the Treatment of Triple-Negative Breast Cancer: A Literature Review. Cureus.

[4] Giaquinto A.N., Sung H., Newman L.A., Freedman R.A., Smith R.A., Star J., Jemal A., Siegel R.L. (2024). Breast Cancer Statistics 2024. CA Cancer J. Clin..

[5] Jie H., Ma W., Huang C. (2025). Diagnosis, Prognosis, and Treatment of Triple-Negative Breast Cancer: A Review. Breast Cancer Targets Ther..

[6] Dieci M.V., Radosevic-Robin N., Fineberg S., van den Eynden G., Ternes N., Penault-Llorca F., Pruneri G., D’Alfonso T.M., Demaria S., Castaneda C. (2018). Update on Tumor-Infiltrating Lymphocytes (TILs) in Breast Cancer, Including Recommendations to Assess TILs in Residual Disease after Neoadjuvant Therapy and in Carcinoma in Situ: A Report of the International Immuno-Oncology Biomarker Working Group on Breast Cancer. Semin. Cancer Biol..

[7] He Y., Jiang Z., Chen C., Wang X. (2018). Classification of Triple-Negative Breast Cancers Based on Immunogenomic Profiling. J. Exp. Clin. Cancer Res..

[8] Medina A., Carballo J., González-Marcano E., Blanca I., Convit A.F. (2024). Breast Cancer Immunotherapy: Realities and Advances. Cancer Innov..

[9] Sahu M., Suryawanshi H. (2021). Immunotherapy: The Future of Cancer Treatment. J. Oral Maxillofac. Pathol. JOMFP.

[10] Riley R.S., June C.H., Langer R., Mitchell M.J. (2019). Delivery Technologies for Cancer Immunotherapy. Nat. Rev. Drug Discov..

[11] Han J., Gao S., Li M., Li X. (2025). Efficacy and Safety of Immunotherapy Combined with Chemotherapy in Both Neoadjuvant and Adjuvant Settings among Triple-Negative Breast Cancer: A Meta-Analysis of Randomized Clinical Trials. Curr. Probl. Cancer.

[12] Ahmadi R., Sadeghi K.F., Sharmsar O., Bagheri Y., Sadeghi K.F., Koochehloghmani H., Hoseinpourfeyzi M. (2025). Recent Findings on the PD-1/PD-L1 Axis in Breast Cancer: Molecular Mechanisms, Immunotherapeutic Potentials, and Clinical Implications. Crit. Rev. Oncol. Hematol..

[13] Essalihi A., Bouchra O., Khadiri K., Khadrouf Z., Karkouri M. (2025). Immunotherapy for Triple-Negative Breast Cancer: Current Trends and Future Prospects. J. Egypt. Natl. Cancer Inst..

[14] Salgado R., Denkert C., Demaria S., Sirtaine N., Klauschen F., Pruneri G., Wienert S., Van Den Eynden G., Baehner F.L., Penault-Llorca F. (2015). The Evaluation of Tumor-Infiltrating Lymphocytes (TILs) in Breast Cancer: Recommendations by an International TILs Working Group 2014. Ann. Oncol..

[15] Alkhayyal N., Elemam N.M., Hussein A., Magdub S., Jundi M., Maghazachi A.A., Talaat I.M., Bendardaf R. (2022). Expression of Immune Checkpoints (PD-L1 and IDO) and Tumour-Infiltrating Lymphocytes in Breast Cancer. Heliyon.

[16] Bertucci F., Finetti P., Mamessier E., Pantaleo M.A., Astolfi A., Ostrowski J., Birnbaum D. (2015). PDL1 Expression Is an Independent Prognostic Factor in Localized GIST. OncoImmunology.

[17] Yang Y., Wang W. (2025). Recent Progress in Immune Evasion Mechanisms of Triple-Negative Breast Cancer. J. Transl. Med..

[18] Cortes J., Lipatov O., Im S.-A., Goncalves A., Lee K.S., Schmid P., Tamura K., Testa L., Ohtani S., Harbeck N. (2025). Association of Potential Biomarkers with Clinical Outcomes in Metastatic Triple-Negative Breast Cancer Treated with Pembrolizumab or Chemotherapy. NPJ Breast Cancer.

[19] Ilieva N., Pencheva M., Hadzhiev H., Tashkova D., Daskalova E., Georgiev P., Genova S. (2024). Impact of Neoadjuvant Therapy on PD-L1 Expression in Triple-Negative Breast Cancer and Correlation with Clinicopathological Factors. Diagnostics.

[20] Diggs L.P., Hsueh E.C. (2017). Utility of PD-L1 Immunohistochemistry Assays for Predicting PD-1/PD-L1 Inhibitor Response. Biomark. Res..

[21] Das R., Deb S., Suresh P.K. (2025). TMB as a Predictive Biomarker for ICI Response in TNBC: Current Evidence and Future Directions for Augmented Anti-Tumor Responses. Clin. Exp. Med..

[22] Santa-Maria C.A., O’Donnell M., Nunes R., Wright J.L., Stearns V. (2022). Integrating Immunotherapy in Early-Stage Triple-Negative Breast Cancer: Practical Evidence-Based Considerations. J. Natl. Compr. Canc. Netw..

[23] Cortes J., Rugo H.S., Cescon D.W., Im S.-A., Yusof M.M., Gallardo C., Lipatov O., Barrios C.H., Perez-Garcia J., Iwata H. (2022). Pembrolizumab plus Chemotherapy in Advanced Triple-Negative Breast Cancer. N. Engl. J. Med..

[24] Wang C., Zhang J., Wang Y., Ouyang T., Li J., Wang T., Fan Z., Fan T., Lin B., Xie Y. (2015). Prevalence of BRCA1 Mutations and Responses to Neoadjuvant Chemotherapy among BRCA1 Carriers and Non-Carriers with Triple-Negative Breast Cancer. Ann. Oncol..

[25] Pohl-Rescigno E., Hauke J., Loibl S., Möbus V., Denkert C., Fasching P.A., Kayali M., Ernst C., Weber-Lassalle N., Hanusch C. (2020). Association of Germline Variant Status with Therapy Response in High-Risk Early-Stage Breast Cancer: A Secondary Analysis of the GeparOcto Randomized Clinical Trial. JAMA Oncol..

[26] Sun S., Liu L., Zhang J., Sun L., Shu W., Yang Z., Yao H., Zhang Z. (2025). The Role of Neoantigens and Tumor Mutational Burden in Cancer Immunotherapy: Advances, Mechanisms, and Perspectives. J. Hematol. Oncol..

[27] Fan Y., He S. (2022). The Characteristics of Tumor Microenvironment in Triple Negative Breast Cancer. Cancer Manag. Res..

[28] Adams S., Gatti-Mays M.E., Kalinsky K., Korde L.A., Sharon E., Amiri-Kordestani L., Bear H., McArthur H.L., Frank E., Perlmutter J. (2019). Current Landscape of Immunotherapy in Breast Cancer: A Review. JAMA Oncol..

[29] Li L., Zhang F., Liu Z., Fan Z. (2023). Immunotherapy for Triple-Negative Breast Cancer: Combination Strategies to Improve Outcome. Cancers.

[30] Ciurescu S., Buciu V., Șerban D., Borozan F., Tomescu L., Cobec I.M., Ilaș D.G., Sas I. (2025). Role of Cytokines in Breast Cancer: A Systematic Review and Meta-Analysis. Biomedicines.

[31] Sabit H., Adel A., Abdelfattah M.M., Ramadan R.M., Nazih M., Abdel-Ghany S., El-hashash A., Arneth B. (2025). The Role of Tumor Microenvironment and Immune Cell Crosstalk in Triple-Negative Breast Cancer (TNBC): Emerging Therapeutic Opportunities. Cancer Lett..

[32] Serrano García L., Jávega B., Llombart Cussac A., Gión M., Pérez-García J.M., Cortés J., Fernández-Murga M.L. (2024). Patterns of Immune Evasion in Triple-Negative Breast Cancer and New Potential Therapeutic Targets: A Review. Front. Immunol..

[33] Meng L., Wu H., Wu J., Ding P., He J., Sang M., Liu L. (2024). Mechanisms of Immune Checkpoint Inhibitors: Insights into the Regulation of Circular RNAS Involved in Cancer Hallmarks. Cell Death Dis..

[34] Tocheva A.S., Mor A. (2017). Checkpoint Inhibitors: Applications for Autoimmunity. Curr. Allergy Asthma Rep..

[35] Hussein O.J., Rayan M., Matarid T.R., Elkhalifa D., Abunada H.H., Therachiyil L., Khalil A., Uddin S., Maccalli C., Korashy H.M. (2025). The Role of Immune Checkpoints in Modulating Cancer Stem Cells Anti-Tumor Immune Responses: Implications and Perspectives in Cancer Therapy. J. Exp. Clin. Cancer Res..

[36] Buchbinder E.I., Desai A. (2016). CTLA-4 and PD-1 Pathways: Similarities, Differences, and Implications of Their Inhibition. Am. J. Clin. Oncol..

[37] Farshbafnadi M., Pastaki Khoshbin A., Rezaei N. (2021). Immune Checkpoint Inhibitors for Triple-Negative Breast Cancer: From Immunological Mechanisms to Clinical Evidence. Int. Immunopharmacol..

[38] Chen H., Yang H., Guo L., Sun Q. (2025). The Role of Immune Checkpoint Inhibitors in Cancer Therapy: Mechanism and Therapeutic Advances. MedComm.

[39] Tang Q., Chen Y., Li X., Long S., Shi Y., Yu Y., Wu W., Han L., Wang S. (2022). The Role of PD-1/PD-L1 and Application of Immune-Checkpoint Inhibitors in Human Cancers. Front. Immunol..

[40] Emens L.A., Cruz C., Eder J.P., Braiteh F., Chung C., Tolaney S.M., Kuter I., Nanda R., Cassier P.A., Delord J.-P. (2019). Long-Term Clinical Outcomes and Biomarker Analyses of Atezolizumab Therapy for Patients with Metastatic Triple-Negative Breast Cancer: A Phase 1 Study. JAMA Oncol..

[41] Hargadon K.M., Johnson C.E., Williams C.J. (2018). Immune Checkpoint Blockade Therapy for Cancer: An Overview of FDA-Approved Immune Checkpoint Inhibitors. Int. Immunopharmacol..

[42] Bao Q., Chen H., Wu S., Tian Z., Tang H. (2025). Role of Immune Checkpoint Inhibitors in Breast Cancer and Hematological Malignancies. Explor. Immunol..

[43] Liu J., Chen Z., Li Y., Zhao W., Wu J., Zhang Z. (2021). PD-1/PD-L1 Checkpoint Inhibitors in Tumor Immunotherapy. Front. Pharmacol..

[44] Kumar S., Chatterjee M., Ghosh P., Ganguly K.K., Basu M., Ghosh M.K. (2022). Targeting PD-1/PD-L1 in Cancer Immunotherapy: An Effective Strategy for Treatment of Triple-Negative Breast Cancer (TNBC) Patients. Genes Dis..

[45] Francisco L.M., Sage P.T., Sharpe A.H. (2010). The PD-1 Pathway in Tolerance and Autoimmunity. Immunol. Rev..

[46] Vuletic A., Mirjacic Martinovic K., Jurisic V. (2025). The Role of Tumor Microenvironment in Triple-Negative Breast Cancer and Its Therapeutic Targeting. Cells.

[47] Zhu Y., Zhu X., Tang C., Guan X., Zhang W. (2021). Progress and Challenges of Immunotherapy in Triple-Negative Breast Cancer. Biochim. Biophys. Acta BBA Rev. Cancer.

[48] Xu-Monette Z.Y., Zhang M., Li J., Young K.H. (2017). PD-1/PD-L1 Blockade: Have We Found the Key to Unleash the Antitumor Immune Response?. Front. Immunol..

[49] Zhu Y., Zhang H., Pan C., He G., Cui X., Yu X., Zhang X., Wu D., Yang J., Wu X. (2023). Integrated Tumor Genomic and Immune Microenvironment Analysis Identifies Predictive Biomarkers Associated with the Efficacy of Neoadjuvant Therapy for Triple-Negative Breast Cancer. Cancer Med..

[50] Wang X., Wang L., Liu Y. (2025). Current Status of Immune Checkpoint Inhibitors and Treatment Responsive Biomarkers for Triple-Negative Breast Cancer. Thorac. Cancer.

[51] Dill E.A., Gru A.A., Atkins K.A., Friedman L.A., Moore M.E., Bullock T.N., Cross J.V., Dillon P.M., Mills A.M. (2017). PD-L1 Expression and Intratumoral Heterogeneity Across Breast Cancer Subtypes and Stages: An Assessment of 245 Primary and 40 Metastatic Tumors. Am. J. Surg. Pathol..

[52] Im S.-A., Cortes J., Cescon D.W., Yusof M.M., Iwata H., Masuda N., Takano T., Huang C.-S., Chung C.-F., Tsugawa K. (2024). Results from the Randomized KEYNOTE-355 Study of Pembrolizumab plus Chemotherapy for Asian Patients with Advanced TNBC. NPJ Breast Cancer.

[53] Liu Z., Jiang Z., Gao Y., Wang L., Chen C., Wang X. (2019). TP53 Mutations Promote Immunogenic Activity in Breast Cancer. J. Oncol..

[54] Yan X., Lv Q., Wu J., Fang J., Peng L., Zhao X. (2025). The Efficacy and Immune-Mediated Safety of PD-1/PD-L1 Combined with Neoadjuvant Chemotherapy in Triple-Negative Breast Cancer: A Meta-Analysis. Front. Oncol..

[55] Schmid P., Salgado R., Park Y.H., Muñoz-Couselo E., Kim S.B., Sohn J., Im S.-A., Foukakis T., Kuemmel S., Dent R. (2020). Pembrolizumab plus Chemotherapy as Neoadjuvant Treatment of High-Risk, Early-Stage Triple-Negative Breast Cancer: Results from the Phase 1b Open-Label, Multicohort KEYNOTE-173 Study. Ann. Oncol..

[56] Schmid P., Cortes J., Dent R., McArthur H., Pusztai L., Kümmel S., Denkert C., Park Y.H., Hui R., Harbeck N. (2024). Overall Survival with Pembrolizumab in Early-Stage Triple-Negative Breast Cancer. N. Engl. J. Med..

[57] Wang Z., You P., Yang Z., Xiao H., Tang X., Pan Y., Li X., Gao F. (2024). PD-1/PD-L1 Immune Checkpoint Inhibitors in the Treatment of Unresectable Locally Advanced or Metastatic Triple Negative Breast Cancer: A Meta-Analysis on Their Efficacy and Safety. BMC Cancer.

[58] Alva A.S., Mangat P.K., Garrett-Mayer E., Halabi S., Hansra D., Calfa C.J., Khalil M.F., Ahn E.R., Cannon T.L., Crilley P. (2021). Pembrolizumab in Patients with Metastatic Breast Cancer with High Tumor Mutational Burden: Results from the Targeted Agent and Profiling Utilization Registry (TAPUR) Study. J. Clin. Oncol..

[59] Heeke A.L., Tan A.R. (2021). Checkpoint Inhibitor Therapy for Metastatic Triple-Negative Breast Cancer. Cancer Metastasis Rev..

[60] Khan M., Du K., Ai M., Wang B., Lin J., Ren A., Chen C., Huang Z., Qiu W., Yuan Y. (2023). PD-L1 Expression as Biomarker of Efficacy of PD-1/PD-L1 Checkpoint Inhibitors in Metastatic Triple Negative Breast Cancer: A Systematic Review and Meta-Analysis. Front. Immunol..

[61] Adams S., Loi S., Toppmeyer D., Cescon D.W., De Laurentiis M., Nanda R., Winer E.P., Mukai H., Tamura K., Armstrong A. (2019). Pembrolizumab Monotherapy for Previously Untreated, PD-L1-Positive, Metastatic Triple-Negative Breast Cancer: Cohort B of the Phase II KEYNOTE-086 Study. Ann. Oncol..

[62] Adams S., Schmid P., Rugo H.S., Winer E.P., Loirat D., Awada A., Cescon D.W., Iwata H., Campone M., Nanda R. (2019). Pembrolizumab Monotherapy for Previously Treated Metastatic Triple-Negative Breast Cancer: Cohort A of the Phase II KEYNOTE-086 Study. Ann. Oncol..

[63] Winer E.P., Lipatov O., Im S.-A., Goncalves A., Muñoz-Couselo E., Lee K.S., Schmid P., Tamura K., Testa L., Witzel I. (2021). Pembrolizumab versus Investigator-Choice Chemotherapy for Metastatic Triple-Negative Breast Cancer (KEYNOTE-119): A Randomised, Open-Label, Phase 3 Trial. Lancet Oncol..

[64] Lee J., Yoo J.-S., Kim J.H., Lee D.Y., Yang K., Kim B., Choi J.-I., Jang J.W., Choi J.Y., Yoon S.K. (2024). Prognostic Significance of Combined PD-L1 Expression in Malignant and Infiltrating Cells in Hepatocellular Carcinoma Treated with Atezolizumab and Bevacizumab. Front. Immunol..

[65] Schmid P., Cortes J., Pusztai L., McArthur H., Kümmel S., Bergh J., Denkert C., Park Y.H., Hui R., Harbeck N. (2020). Pembrolizumab for Early Triple-Negative Breast Cancer. N. Engl. J. Med..

[66] Dirix L.Y., Takacs I., Jerusalem G., Nikolinakos P., Arkenau H.-T., Forero-Torres A., Boccia R., Lippman M.E., Somer R., Smakal M. (2018). Avelumab, an Anti-PD-L1 Antibody, in Patients with Locally Advanced or Metastatic Breast Cancer: A Phase 1b JAVELIN Solid Tumor Study. Breast Cancer Res. Treat..

[67] Nanda R., Chow L.Q.M., Dees E.C., Berger R., Gupta S., Geva R., Pusztai L., Pathiraja K., Aktan G., Cheng J.D. (2016). Pembrolizumab in Patients with Advanced Triple-Negative Breast Cancer: Phase Ib KEYNOTE-012 Study. J. Clin. Oncol..

[68] Dos Santos A.L.S., Da Silva J.L., De Albuquerque L.Z., Neto A.L.A., Da Silva C.F., Cerva L.A.M., Small I.A., Rodrigues F.R., De Macedo F.C., Marcelino C.P. (2025). Unveiling the Landscape of PD-L1 Expression and Tumor-Infiltrating Lymphocyte Subtypes in Advanced Triple-Negative Breast Cancer in Brazil. Breast Cancer Targets Ther..

[69] Zhang M., Sun H., Zhao S., Wang Y., Pu H., Wang Y., Zhang Q. (2017). Expression of PD-L1 and Prognosis in Breast Cancer: A Meta-Analysis. Oncotarget.

[70] Rugo H.S., Loi S., Adams S., Schmid P., Schneeweiss A., Barrios C.H., Iwata H., Diéras V., Winer E.P., Kockx M.M. (2021). PD-L1 Immunohistochemistry Assay Comparison in Atezolizumab Plus Nab -Paclitaxel–Treated Advanced Triple-Negative Breast Cancer. JNCI J. Natl. Cancer Inst..

[71] Büttner R., Gosney J.R., Skov B.G., Adam J., Motoi N., Bloom K.J., Dietel M., Longshore J.W., López-Ríos F., Penault-Llorca F. (2017). Programmed Death-Ligand 1 Immunohistochemistry Testing: A Review of Analytical Assays and Clinical Implementation in Non–Small-Cell Lung Cancer. J. Clin. Oncol..

[72] Chae Y.K., Pan A., Davis A.A., Raparia K., Mohindra N.A., Matsangou M., Giles F.J. (2016). Biomarkers for PD-1/PD-L1 Blockade Therapy in Non–Small-Cell Lung Cancer: Is PD-L1 Expression a Good Marker for Patient Selection?. Clin. Lung Cancer.

[73] Zouein J., Kesrouani C., Kourie H.R. (2021). PD-L1 Expression as a Predictive Biomarker for Immune Checkpoint Inhibitors: Between a Dream and a Nightmare. Immunotherapy.

[74] Yanagihara K., Nagata K., Yamakawa T., Kato S., Tamura M., Yoshida M. (2025). Decline of PD-L1 Immunoreactivity with Storage Duration in Formalin-Fixed Paraffin-Embedded Breast Cancer Specimens: Implications for Diagnostic Accuracy and Immunotherapy Eligibility in Triple-Negative Breast Cancer. Cancers.

[75] Pang J.-M.B., Castles B., Byrne D.J., Button P., Hendry S., Lakhani S.R., Sivasubramaniam V., Cooper W.A., Armes J., Millar E.K.A. (2021). SP142 PD-L1 Scoring Shows High Interobserver and Intraobserver Agreement in Triple-Negative Breast Carcinoma But Overall Low Percentage Agreement With Other PD-L1 Clones SP263 and 22C3. Am. J. Surg. Pathol..

[76] Badve S.S., Penault-Llorca F., Reis-Filho J.S., Deurloo R., Siziopikou K.P., D’Arrigo C., Viale G. (2022). Determining PD-L1 Status in Patients with Triple-Negative Breast Cancer: Lessons Learned from IMpassion130. JNCI J. Natl. Cancer Inst..

[77] Rodrigues A., Nogueira C., Marinho L.C., Velozo G., Sousa J., Silva P.G., Tavora F. (2022). Computer-Assisted Tumor Grading, Validation of PD-L1 Scoring, and Quantification of CD8-Positive Immune Cell Density in Urothelial Carcinoma, a Visual Guide for Pathologists Using QuPath. Surg. Exp. Pathol..

[78] Solinas C., Carbognin L., De Silva P., Criscitiello C., Lambertini M. (2017). Tumor-Infiltrating Lymphocytes in Breast Cancer According to Tumor Subtype: Current State of the Art. Breast.

[79] Kraja F.P., Jurisic V.B., Hromić-Jahjefendić A., Rossopoulou N., Katsila T., Mirjacic Martinovic K., De Las Rivas J., Diaconu C.C., Szöőr Á. (2025). Tumor-Infiltrating Lymphocytes in Cancer Immunotherapy: From Chemotactic Recruitment to Translational Modeling. Front. Immunol..

[80] Arora S., Velichinskii R., Lesh R.W., Ali U., Kubiak M., Bansal P., Borghaei H., Edelman M.J., Boumber Y. (2019). Existing and Emerging Biomarkers for Immune Checkpoint Immunotherapy in Solid Tumors. Adv. Ther..

[81] Hamada T., Soong T.R., Masugi Y., Kosumi K., Nowak J.A., da Silva A., Mu X.J., Twombly T.S., Koh H., Yang J. (2018). TIME (Tumor Immunity in the MicroEnvironment) Classification Based on Tumor CD274 (PD-L1) Expression Status and Tumor-Infiltrating Lymphocytes in Colorectal Carcinomas. OncoImmunology.

[82] Denkert C., von Minckwitz G., Darb-Esfahani S., Lederer B., Heppner B.I., Weber K.E., Budczies J., Huober J., Klauschen F., Furlanetto J. (2018). Tumour-Infiltrating Lymphocytes and Prognosis in Different Subtypes of Breast Cancer: A Pooled Analysis of 3771 Patients Treated with Neoadjuvant Therapy. Lancet Oncol..

[83] de Moraes F.C.A., Souza M.E.C., Sano V.K.T., Moraes R.A., Melo A.C. (2025). Association of Tumor-Infiltrating Lymphocytes with Clinical Outcomes in Patients with Triple-Negative Breast Cancer Receiving Neoadjuvant Chemotherapy: A Systematic Review and Meta-Analysis. Clin. Transl. Oncol..

[84] Finkelman B.S., Zhang H., Hicks D.G., Rimm D.L., Turner B.M. (2025). Tumor Infiltrating Lymphocytes in Breast Cancer: A Narrative Review with Focus on Analytic Validity, Clinical Validity, and Clinical Utility. Hum. Pathol..

[85] Cai X., Chen Y., Li Q., Wei T., Yang Y., Ye R., Chao X., Li M., He J., Luo R. (2025). Prognostic Value of Tumor-Infiltrating Lymphocytes (TILs) in Luminal Breast Cancer: A Novel Computational Method for Assessing TILs Abundance and Spatial Distribution Patterns. Breast Edinb. Scotl..

[86] Emens L.A., Molinero L., Loi S., Rugo H.S., Schneeweiss A., Diéras V., Iwata H., Barrios C.H., Nechaeva M., Nguyen-Duc A. (2021). Atezolizumab and Nab -Paclitaxel in Advanced Triple-Negative Breast Cancer: Biomarker Evaluation of the IMpassion130 Study. JNCI J. Natl. Cancer Inst..

[87] Yarchoan M., Albacker L.A., Hopkins A.C., Montesion M., Murugesan K., Vithayathil T.T., Zaidi N., Azad N.S., Laheru D.A., Frampton G.M. (2019). PD-L1 Expression and Tumor Mutational Burden Are Independent Biomarkers in Most Cancers. JCI Insight.

[88] Goodman A.M., Kato S., Bazhenova L., Patel S.P., Frampton G.M., Miller V., Stephens P.J., Daniels G.A., Kurzrock R. (2017). Tumor Mutational Burden as an Independent Predictor of Response to Immunotherapy in Diverse Cancers. Mol. Cancer Ther..

[89] Han Y., Rovella V., Smirnov A., Buonomo O.C., Mauriello A., Perretta T., Shi Y., Woodmsith J., Bischof J., TOR CENTRE (2023). A BRCA2 Germline Mutation and High Expression of Immune Checkpoints in a TNBC Patient. Cell Death Discov..

[90] Barroso-Sousa R., Keenan T.E., Pernas S., Exman P., Jain E., Garrido-Castro A.C., Hughes M., Bychkovsky B., Umeton R., Files J.L. (2020). Tumor Mutational Burden and PTEN Alterations as Molecular Correlates of Response to PD-1/L1 Blockade in Metastatic Triple-Negative Breast Cancer. Clin. Cancer Res..

[91] Shah S.N., Hile S.E., Eckert K.A. (2010). Defective Mismatch Repair, Microsatellite Mutation Bias, and Variability in Clinical Cancer Phenotypes. Cancer Res..

[92] Xu Y., Fu Y., Zhu B., Wang J., Zhang B. (2020). Predictive Biomarkers of Immune Checkpoint Inhibitors-Related Toxicities. Front. Immunol..

[93] Ren X., Song Y., Wang J., Chen L., Pang J., Zhou L., Shen S., Cao X., Wang Y., Shao M. (2021). Mismatch Repair Deficiency and Microsatellite Instability in Triple-Negative Breast Cancer: A Retrospective Study of 440 Patients. Front. Oncol..

[94] Li Y., Wang J., Wu L., Li X., Zhang X., Zhang G., Xu S., Sun S., Jiao S. (2021). Diversity of Dominant Peripheral T Cell Receptor Clone and Soluble Immune Checkpoint Proteins Associated with Clinical Outcomes Following Immune Checkpoint Inhibitor Treatment in Advanced Cancers. Front. Immunol..

[95] Kurata K., Kubo M., Kai M., Mori H., Kawaji H., Kaneshiro K., Yamada M., Nishimura R., Osako T., Arima N. (2020). Microsatellite Instability in Japanese Female Patients with Triple-Negative Breast Cancer. Breast Cancer.

[96] Kim G.-R., Choi J.-M. (2022). Current Understanding of Cytotoxic T Lymphocyte Antigen-4 (CTLA-4) Signaling in T-Cell Biology and Disease Therapy. Mol. Cells.

[97] Linsley P.S., Greene J.L., Brady W., Bajorath J., Ledbetter J.A., Peach R. (1994). Human B7-1 (CD80) and B7-2 (CD86) Bind with Similar Avidities but Distinct Kinetics to CD28 and CTLA-4 Receptors. Immunity.

[98] Lotze M.T., Olejniczak S.H., Skokos D. (2024). CD28 Co-Stimulation: Novel Insights and Applications in Cancer Immunotherapy. Nat. Rev. Immunol..

[99] Gaikwad S., Agrawal M.Y., Kaushik I., Ramachandran S., Srivastava S.K. (2022). Immune Checkpoint Proteins: Signaling Mechanisms and Molecular Interactions in Cancer Immunotherapy. Semin. Cancer Biol..

[100] Wang X., He J., Ding G., Tang Y., Wang Q. (2025). Overcoming Resistance to PD-1 and CTLA-4 Blockade Mechanisms and Therapeutic Strategies. Front. Immunol..

[101] Luo C., Wang P., He S., Zhu J., Shi Y., Wang J. (2022). Progress and Prospect of Immunotherapy for Triple-Negative Breast Cancer. Front. Oncol..

[102] Yi H., Li Y., Tan Y., Fu S., Tang F., Deng X. (2021). Immune Checkpoint Inhibition for Triple-Negative Breast Cancer: Current Landscape and Future Perspectives. Front. Oncol..

[103] Choi J., Lee S.Y. (2020). Clinical Characteristics and Treatment of Immune-Related Adverse Events of Immune Checkpoint Inhibitors. Immune Netw..

[104] Ramos-Casals M., Brahmer J.R., Callahan M.K., Flores-Chávez A., Keegan N., Khamashta M.A., Lambotte O., Mariette X., Prat A., Suárez-Almazor M.E. (2020). Immune-Related Adverse Events of Checkpoint Inhibitors. Nat. Rev. Dis. Primer.

[105] Dalvin L.A., Shields C.L., Orloff M., Sato T., Shields J.A. (2018). CHECKPOINT INHIBITOR IMMUNE THERAPY: Systemic Indications and Ophthalmic Side Effects. Retina.

[106] Quiruz L., Yavari N., Kikani B., Gupta A.S., Wai K.M., Kossler A.L., Ludwig C., Koo E.B., Rahimy E., Mruthyunjaya P. (2024). Ophthalmic Immune-Related Adverse Events and Association with Survival: Results from a Real-World Database. Am. J. Ophthalmol..

[107] Brahmer J.R., Lacchetti C., Schneider B.J., Atkins M.B., Brassil K.J., Caterino J.M., Chau I., Ernstoff M.S., Gardner J.M., Ginex P. (2018). Management of Immune-Related Adverse Events in Patients Treated with Immune Checkpoint Inhibitor Therapy: American Society of Clinical Oncology Clinical Practice Guideline. J. Clin. Oncol..

[108] Morgado M., Plácido A., Morgado S., Roque F. (2020). Management of the Adverse Effects of Immune Checkpoint Inhibitors. Vaccines.

[109] Qian J., Liu Y. (2025). Recent Advances in Adoptive Cell Therapy for Cancer Immunotherapy. Front. Immunol..

[110] Zafar A., Khatoon S., Khan M.J., Abu J., Naeem A. (2025). Advancements and Limitations in Traditional Anti-Cancer Therapies: A Comprehensive Review of Surgery, Chemotherapy, Radiation Therapy, and Hormonal Therapy. Discov. Oncol..

[111] Liu Z., Yang C. (2016). A Mathematical Model of Cancer Treatment by Radiotherapy Followed by Chemotherapy. Math. Comput. Simul..

[112] van Diepen A.E., Kucukcelebi S., de Vos-Geelen J., Kooreman N.G. (2026). Immunotherapy for Solid Tumours: Current Clinical Landscape and Future Directions. Eur. Surg. Res..

[113] Zhang M., Liu C., Tu J., Tang M., Ashrafizadeh M., Nabavi N., Sethi G., Zhao P., Liu S. (2025). Advances in Cancer Immunotherapy: Historical Perspectives, Current Developments, and Future Directions. Mol. Cancer.

[114] Nasiri F., Kazemi M., Mirarefin S.M.J., Mahboubi Kancha M., Ahmadi Najafabadi M., Salem F., Dashti Shokoohi S., Evazi Bakhshi S., Safarzadeh Kozani P., Safarzadeh Kozani P. (2022). CAR-T Cell Therapy in Triple-Negative Breast Cancer: Hunting the Invisible Devil. Front. Immunol..

[115] Zugasti I., Espinosa-Aroca L., Fidyt K., Mulens-Arias V., Diaz-Beya M., Juan M., Urbano-Ispizua Á., Esteve J., Velasco-Hernandez T., Menéndez P. (2025). CAR-T Cell Therapy for Cancer: Current Challenges and Future Directions. Signal Transduct. Target. Ther..

[116] Ayala Ceja M., Khericha M., Harris C.M., Puig-Saus C., Chen Y.Y. (2024). CAR-T Cell Manufacturing: Major Process Parameters and next-Generation Strategies. J. Exp. Med..

[117] Sadelain M., Brentjens R., Rivière I. (2013). The Basic Principles of Chimeric Antigen Receptor Design. Cancer Discov..

[118] Mao R., Kong W., He Y. (2022). The Affinity of Antigen-Binding Domain on the Antitumor Efficacy of CAR T Cells: Moderate Is Better. Front. Immunol..

[119] Dees S., Ganesan R., Singh S., Grewal I.S. (2020). Emerging CAR-T Cell Therapy for the Treatment of Triple-Negative Breast Cancer. Mol. Cancer Ther..

[120] Larson R.C., Maus M.V. (2021). Recent Advances and Discoveries in the Mechanisms and Functions of CAR T Cells. Nat. Rev. Cancer.

[121] Rossig C. (2018). CAR T Cell Immunotherapy in Hematology and Beyond. Clin. Immunol..

[122] Chmielewski M., Kopecky C., Hombach A.A., Abken H. (2011). IL-12 Release by Engineered T Cells Expressing Chimeric Antigen Receptors Can Effectively Muster an Antigen-Independent Macrophage Response on Tumor Cells That Have Shut down Tumor Antigen Expression. Cancer Res..

[123] Sheikh M., Madia D., Telrandhe U.B., Bagga H., Deshmukh A. (2025). Advancing Breast Cancer Treatment through Dual Targeting CAR T Cell Therapy. Discov. Oncol..

[124] Chen X., Habib S., Alexandru M., Chauhan J., Evan T., Troka J.M., Rahimi A., Esapa B., Tull T.J., Ng W.Z. (2024). Chondroitin Sulfate Proteoglycan 4 (CSPG4) as an Emerging Target for Immunotherapy to Treat Melanoma. Cancers.

[125] Wang X., Osada T., Wang Y., Yu L., Sakakura K., Katayama A., McCarthy J.B., Brufsky A., Chivukula M., Khoury T. (2010). CSPG4 Protein as a New Target for the Antibody-Based Immunotherapy of Triple-Negative Breast Cancer. JNCI J. Natl. Cancer Inst..

[126] Amoury M., Mladenov R., Nachreiner T., Pham A.-T., Hristodorov D., Di Fiore S., Helfrich W., Pardo A., Fey G., Schwenkert M. (2016). A Novel Approach for Targeted Elimination of CSPG4-Positive Triple-Negative Breast Cancer Cells Using a MAP Tau-Based Fusion Protein. Int. J. Cancer.

[127] Bui T.M., Wiesolek H.L., Sumagin R. (2020). ICAM-1: A Master Regulator of Cellular Responses in Inflammation, Injury Resolution, and Tumorigenesis. J. Leukoc. Biol..

[128] Guo P., Huang J., Wang L., Jia D., Yang J., Dillon D.A., Zurakowski D., Mao H., Moses M.A., Auguste D.T. (2014). ICAM-1 as a Molecular Target for Triple Negative Breast Cancer. Proc. Natl. Acad. Sci. USA.

[129] Curio S., Jonsson G., Marinović S. (2021). A Summary of Current NKG2D-Based CAR Clinical Trials. Immunother. Adv..

[130] Saliu M.A., Wang Q., Salisu M.D., Ren Y., Zhang P., Suleiman R.B., Cao B., Xu Y., Liu X., Lluis F. (2025). Mesothelin-Targeted CAR-T Cells Secreting NKG2D-BiTEs Exhibit Potent Efficacy against Triple-Negative Breast Cancer. Exp. Hematol. Oncol..

[131] Zhang T., Lemoi B.A., Sentman C.L. (2005). Chimeric NK-Receptor-Bearing T Cells Mediate Antitumor Immunotherapy. Blood.

[132] Marei H.E., Bedair K., Hasan A., Al-Mansoori L., Caratelli S., Sconocchia G., Gaiba A., Cenciarelli C. (2025). Current Status and Innovative Developments of CAR-T-Cell Therapy for the Treatment of Breast Cancer. Cancer Cell Int..

[133] Getu A.A., Tigabu A., Zhou M., Lu J., Fodstad Ø., Tan M. (2023). New Frontiers in Immune Checkpoint B7-H3 (CD276) Research and Drug Development. Mol. Cancer.

[134] Castellanos J.R., Purvis I.J., Labak C.M., Guda M.R., Tsung A.J., Velpula K.K., Asuthkar S. (2017). B7-H3 Role in the Immune Landscape of Cancer. Am. J. Clin. Exp. Immunol..

[135] Guo Y., Wang X., Zhang C., Chen W., Fu Y., Yu Y., Chen Y., Shao T., Zhang J., Ding G. (2025). Tumor Immunotherapy Targeting B7-H3: From Mechanisms to Clinical Applications. ImmunoTargets Ther..

[136] Rehorst P., Kros A. (2025). A General Logic-Gating Framework for CAR-T and Nanocarrier Cancer Therapies. J. Control. Release.

[137] Zhu J., Zhou J., Tang Y., Huang R., Lu C., Qian K., Zhou Q., Zhang J., Yang X., Zhou W. (2025). Advancements and Challenges in CAR-T Cell Therapy for Solid Tumors: A Comprehensive Review of Antigen Targets, Strategies, and Future Directions. Cancer Cell Int..

[138] Corti C., Koca B., Rahman T., Mittendorf E.A., Tolaney S.M. (2025). Recent Advances in Immune Checkpoint Inhibitors for Triple-Negative Breast Cancer. ImmunoTargets Ther..

[139] Zhang X., Ge X., Jiang T., Yang R., Li S. (2022). Research Progress on Immunotherapy in Triple-negative Breast Cancer. Int. J. Oncol..

[140] Ma S., Li X., Wang X., Cheng L., Li Z., Zhang C., Ye Z., Qian Q. (2019). Current Progress in CAR-T Cell Therapy for Solid Tumors. Int. J. Biol. Sci..

[141] Escobar G., Berger T.R., Maus M.V. (2025). CAR-T Cells in Solid Tumors: Challenges and Breakthroughs. Cell Rep. Med..

[142] Sterner R.C., Sterner R.M. (2021). CAR-T Cell Therapy: Current Limitations and Potential Strategies. Blood Cancer J..

[143] Chen R., Johnson J., Rezazadeh A., Dudek A.Z. (2025). Tumour-Infiltrating Lymphocyte Therapy Landscape: Prospects and Challenges. BMJ Oncol..

[144] Granhøj J.S., Witness Præst Jensen A., Presti M., Met Ö., Svane I.M., Donia M. (2022). Tumor-Infiltrating Lymphocytes for Adoptive Cell Therapy: Recent Advances, Challenges, and Future Directions. Expert Opin. Biol. Ther..

[145] Sikandar B., Qureshi M.A., Naseem S., Khan S., Mirza T. (2017). Increased Tumour Infiltration of CD4+ and CD8+ T-Lymphocytes in Patients with Triple Negative Breast Cancer Suggests Susceptibility to Immune Therapy. Asian Pac. J. Cancer Prev..

[146] Chen Y., Anwar M., Wang X., Zhang B., Ma B. (2025). Integrative Transcriptomic and Single-Cell Analysis Reveals IL27RA as a Key Immune Regulator and Therapeutic Indicator in Breast Cancer. Discov. Oncol..

[147] Oshi M., Asaoka M., Tokumaru Y., Yan L., Matsuyama R., Ishikawa T., Endo I., Takabe K. (2020). CD8 T Cell Score as a Prognostic Biomarker for Triple Negative Breast Cancer. Int. J. Mol. Sci..

[148] Cao G., Ding C., Dai J., Qiu X. (2025). Oncolytic Virus and Immunogenic Cell Death in Cancer Therapy. Tumour Virus Res..

[149] Ghavimi R., Rahimian L., Mohammadi M., Dutta O., Mohan H., Chouljenko V., Omolekan T.O., Chamcheu J.C., Kousoulas K.G. (2025). Viral Warriors: Unlocking the Immune System’s Potential with Oncolytic Viruses in Cancer Immunotherapy. Mol. Ther. Oncol..

[150] Kaufman H.L., Kohlhapp F.J., Zloza A. (2015). Oncolytic Viruses: A New Class of Immunotherapy Drugs. Nat. Rev. Drug Discov..

[151] Cejalvo J.M., Falato C., Villanueva L., Tolosa P., González X., Pascal M., Canes J., Gavilá J., Manso L., Pascual T. (2022). Oncolytic Viruses: A New Immunotherapeutic Approach for Breast Cancer Treatment?. Cancer Treat. Rev..

[152] Sanjuán R., Domingo-Calap P. (2016). Mechanisms of Viral Mutation. Cell. Mol. Life Sci..

[153] O’Bryan S.M., Mathis J.M. (2018). Oncolytic Virotherapy for Breast Cancer Treatment. Curr. Gene Ther..

[154] Holl E.K., Brown M.C., Boczkowski D., McNamara M.A., George D.J., Bigner D.D., Gromeier M., Nair S.K. (2016). Recombinant Oncolytic Poliovirus, PVSRIPO, Has Potent Cytotoxic and Innate Inflammatory Effects, Mediating Therapy in Human Breast and Prostate Cancer Xenograft Models. Oncotarget.

[155] Sugiyama T., Yoneda M., Kuraishi T., Hattori S., Inoue Y., Sato H., Kai C. (2013). Measles Virus Selectively Blind to Signaling Lymphocyte Activation Molecule as a Novel Oncolytic Virus for Breast Cancer Treatment. Gene Ther..

[156] Zhang J., Tai L.-H., Ilkow C.S., Alkayyal A.A., Ananth A.A., de Souza C.T., Wang J., Sahi S., Ly L., Lefebvre C. (2014). Maraba MG1 Virus Enhances Natural Killer Cell Function via Conventional Dendritic Cells to Reduce Postoperative Metastatic Disease. Mol. Ther..

[157] Zhang Y., Nagalo B.M. (2022). Immunovirotherapy Based on Recombinant Vesicular Stomatitis Virus: Where Are We?. Front. Immunol..

[158] Tang S., Yong L., Cui Y., Li H., Bischof E., Cai F. (2025). Harnessing Oncolytic Viruses for Targeted Therapy in Triple-Negative Breast Cancer. Int. J. Med. Sci..

[159] Lin D., Shen Y., Liang T. (2023). Oncolytic Virotherapy: Basic Principles, Recent Advances and Future Directions. Signal Transduct. Target. Ther..

[160] Bahreyni A., Mohamud Y., Luo H. (2024). Oncolytic Virus-Based Combination Therapy in Breast Cancer. Cancer Lett..

[161] Omolekan T.O., Folahan J.T., Tesfay M.Z., Mohan H., Dutta O., Rahimian L., Ferdous K.U., Ghavimi R., Cios A., Beng T.K. (2025). Viral Warfare: Unleashing Engineered Oncolytic Viruses to Outsmart Cancer’s Defenses. Front. Immunol..

[162] Wang Y., Li G., Wang H., Qi Q., Wang X., Lu H. (2025). Targeted Therapeutic Strategies for Nectin-4 in Breast Cancer: Recent Advances and Future Prospects. Breast Edinb. Scotl..

[163] Wu Y.-Y., Sun T.-K., Chen M.-S., Munir M., Liu H.-J. (2023). Oncolytic Viruses-Modulated Immunogenic Cell Death, Apoptosis and Autophagy Linking to Virotherapy and Cancer Immune Response. Front. Cell. Infect. Microbiol..

[164] Ma J., Ramachandran M., Jin C., Quijano-Rubio C., Martikainen M., Yu D., Essand M. (2020). Characterization of Virus-Mediated Immunogenic Cancer Cell Death and the Consequences for Oncolytic Virus-Based Immunotherapy of Cancer. Cell Death Dis..

[165] Prasad V., Greber U.F. (2021). The Endoplasmic Reticulum Unfolded Protein Response—Homeostasis, Cell Death and Evolution in Virus Infections. FEMS Microbiol. Rev..

[166] Zhu W., Zhang H., Shi Y., Song M., Zhu B., Wei L. (2013). Oncolytic Adenovirus Encoding Tumor Necrosis Factor-Related Apoptosis Inducing Ligand (TRAIL) Inhibits the Growth and Metastasis of Triple-Negative Breast Cancer. Cancer Biol. Ther..

[167] Yang Y.-L., Yang F., Huang Z.-Q., Li Y.-Y., Shi H.-Y., Sun Q., Ma Y., Wang Y., Zhang Y., Yang S. (2023). T Cells, NK Cells, and Tumor-Associated Macrophages in Cancer Immunotherapy and the Current State of the Art of Drug Delivery Systems. Front. Immunol..

[168] Spranger S., Dai D., Horton B., Gajewski T.F. (2017). Tumor-Residing Batf3 Dendritic Cells Are Required for Effector T Cell Trafficking and Adoptive T Cell Therapy. Cancer Cell.

[169] Kepp O., Senovilla L., Vitale I., Vacchelli E., Adjemian S., Agostinis P., Apetoh L., Aranda F., Barnaba V., Bloy N. (2014). Consensus Guidelines for the Detection of Immunogenic Cell Death. OncoImmunology.

[170] Huang Z., Xu J., Wang Y., Fu Z., Zhang Y., Zhao Q., Zhang R., Hu X., Cheng X., Hu C. (2026). The Role of MHC Molecules in Cancer Immunotherapy: Insights into Tumor Immune Evasion and Therapeutic Strategies. Crit. Rev. Oncol. Hematol..

[171] Zamarin D., Holmgaard R.B., Ricca J., Plitt T., Palese P., Sharma P., Merghoub T., Wolchok J.D., Allison J.P. (2017). Intratumoral Modulation of the Inducible Co-Stimulator ICOS by Recombinant Oncolytic Virus Promotes Systemic Anti-Tumour Immunity. Nat. Commun..

[172] Jin S., Wang Q., Wu H., Pang D., Xu S. (2021). Oncolytic Viruses for Triple Negative Breast Cancer and Beyond. Biomark. Res..

[173] Ghouse S.M., Nguyen H.-M., Bommareddy P.K., Guz-Montgomery K., Saha D. (2020). Oncolytic Herpes Simplex Virus Encoding IL12 Controls Triple-Negative Breast Cancer Growth and Metastasis. Front. Oncol..

[174] Governa V., Brittoli A., Mele V., Pinamonti M., Terracciano L., Muenst S., Iezzi G., Spagnoli G.C., Zajac P., Trella E. (2019). A Replication-Incompetent CD154/40L Recombinant Vaccinia Virus Induces Direct and Macrophage-Mediated Antitumor Effects in Vitro and in Vivo. OncoImmunology.

[175] Niavarani S.-R., Lawson C., Boudaud M., Simard C., Tai L.-H. (2020). Oncolytic Vesicular Stomatitis Virus–Based Cellular Vaccine Improves Triple-Negative Breast Cancer Outcome by Enhancing Natural Killer and CD8^+^ T-Cell Functionality. J. Immunother. Cancer.

[176] Aravind A., Shaji V., Ahmed M., Dagamajalu S., Vijayakumar M., Prasad T.S.K., Raju R. (2025). An Assembled Molecular Signaling Map of Interleukin-24: A Resource to Decipher Its Multifunctional Immunoregulatory Role in Pathophysiological Conditions. Front. Immunol..

[177] Deng L., Fan J., Ding Y., Yang X., Huang B., Hu Z. (2020). Target Therapy with Vaccinia Virus Harboring IL-24 For Human Breast Cancer. J. Cancer.

[178] Huang Z., Yu P., Tang J. (2020). Characterization of Triple-Negative Breast Cancer MDA-MB-231 Cell Spheroid Model. OncoTargets Ther..

[179] Rajwani J., Vishnevskiy D., Turk M., Naumenko V., Gafuik C., Kim D.-S., Mah L.K., Snelling S., Gonzales G.A., Xue J. (2024). VSV^∆M51^ Drives CD8^+^ T Cell-Mediated Tumour Regression through Infection of Both Cancer and Non-Cancer Cells. Nat. Commun..

[180] Kaufman H.L., Shalhout S.Z., Iodice G. (2022). Talimogene Laherparepvec: Moving from First-In-Class to Best-In-Class. Front. Mol. Biosci..

[181] Tijtgat J., De Munck J., Dufait I., Schwarze J.K., Van Riet I., Franceschini L., Breckpot K., Aerts J.L., Neyns B., Tuyaerts S. (2021). Unraveling the Effects of a Talimogene Laherparepvec (T-VEC)-Induced Tumor Oncolysate on Myeloid Dendritic Cells. Front. Immunol..

[182] Zahavi D., Weiner L. (2020). Monoclonal Antibodies in Cancer Therapy. Antibodies.

[183] Okpasuo O.J., Olaoba O.T., Bokolo P., Aleke A.S., Dare A., Adelusi T.I., Jackson C.D. (2026). The Evolving Landscape of Antibody-Based Cancer Therapies: From Monospecific to Multi-Specific and Beyond. Crit. Rev. Oncol. Hematol..

[184] Nagayama A., Vidula N., Ellisen L., Bardia A. (2020). Novel Antibody–Drug Conjugates for Triple Negative Breast Cancer. Ther. Adv. Med. Oncol..

[185] Wahby S., Fashoyin-Aje L., Osgood C.L., Cheng J., Fiero M.H., Zhang L., Tang S., Hamed S.S., Song P., Charlab R. (2021). FDA Approval Summary: Accelerated Approval of Sacituzumab Govitecan-Hziy for Third-Line Treatment of Metastatic Triple-Negative Breast Cancer. Clin. Cancer Res..

[186] Bagegni N.A., Giridhar K.V., Stewart D. (2025). HER2-Low and HER2-Ultralow Metastatic Breast Cancer and Trastuzumab Deruxtecan: Common Clinical Questions and Answers. Cancers.

[187] Starodub A.N., Ocean A.J., Shah M.A., Guarino M.J., Picozzi V.J., Vahdat L.T., Thomas S.S., Govindan S.V., Maliakal P.P., Wegener W.A. (2015). First-in-Human Trial of a Novel Anti-Trop-2 Antibody-SN-38 Conjugate, Sacituzumab Govitecan, for the Treatment of Diverse Metastatic Solid Tumors. Clin. Cancer Res..

[188] Sakach E., Sacks R., Kalinsky K. (2022). Trop-2 as a Therapeutic Target in Breast Cancer. Cancers.

[189] Lin H., Huang J.-F., Qiu J.-R., Zhang H.-L., Tang X.-J., Li H., Wang C.-J., Wang Z.-C., Feng Z.-Q., Zhu J. (2013). Significantly Upregulated TACSTD2 and Cyclin D1 Correlate with Poor Prognosis of Invasive Ductal Breast Cancer. Exp. Mol. Pathol..

[190] Bardia A., Mayer I.A., Diamond J.R., Moroose R.L., Isakoff S.J., Starodub A.N., Shah N.C., O’Shaughnessy J., Kalinsky K., Guarino M. (2017). Efficacy and Safety of Anti-Trop-2 Antibody Drug Conjugate Sacituzumab Govitecan (IMMU-132) in Heavily Pretreated Patients with Metastatic Triple-Negative Breast Cancer. J. Clin. Oncol..

[191] Wali A.F., El-Tanani M., Talath S., Rabbani S.A., Rangraze I.R., Satyam S.M., Avagimyan A., Hoffmann K., Ilias I., Ispas S. (2025). Antibody-Drug Conjugates and Beyond: Next-Generation Targeted Therapies for Breast Cancer. Cancers.

[192] Voigt W., Matsui S., Yin M.B., Burhans W.C., Minderman H., Rustum Y.M. (1998). Topoisomerase-I Inhibitor SN-38 Can Induce DNA Damage and Chromosomal Aberrations Independent from DNA Synthesis. Anticancer Res..

[193] Goldenberg D.M., Cardillo T.M., Govindan S.V., Rossi E.A., Sharkey R.M. (2015). Trop-2 Is a Novel Target for Solid Cancer Therapy with Sacituzumab Govitecan (IMMU-132), an Antibody-Drug Conjugate (ADC). Oncotarget.

[194] Goldenberg D.M., Sharkey R.M. (2019). Antibody-Drug Conjugates Targeting TROP-2 and Incorporating SN-38: A Case Study of Anti-TROP-2 Sacituzumab Govitecan. mAbs.

[195] Antherieu G., Thibaud V., Sesques P., Jacot W., Menia K., Balducci L., Falandry C. (2025). Neutropenic Events Associated with New Anticancer Treatments, from Pathophysiology to Practice: A Scoping Review-NEW Group (Neutropenic Events Working Group). Support. Care Cancer.

[196] Bardia A., Hurvitz S.A., Tolaney S.M., Loirat D., Punie K., Oliveira M., Brufsky A., Sardesai S.D., Kalinsky K., Zelnak A.B. (2021). Sacituzumab Govitecan in Metastatic Triple-Negative Breast Cancer. N. Engl. J. Med..

[197] Yan S., Sun X., Wang K. (2025). From Cold to Hot Tumors: Feasibility of Applying Therapeutic Insights to TNBC. Discov. Oncol..

[198] Tufail M., Jiang C.-H., Li N. (2025). Immune Evasion in Cancer: Mechanisms and Cutting-Edge Therapeutic Approaches. Signal Transduct. Target. Ther..

[199] Zhou Z., Zhou Q. (2025). Immunotherapy Resistance in Triple-Negative Breast Cancer: Molecular Mechanisms, Tumor Microenvironment, and Therapeutic Implications. Front. Oncol..

[200] Le D.T., Durham J.N., Smith K.N., Wang H., Bartlett B.R., Aulakh L.K., Lu S., Kemberling H., Wilt C., Luber B.S. (2017). Mismatch Repair Deficiency Predicts Response of Solid Tumors to PD-1 Blockade. Science.

[201] Li J., Jia Z., Dong L., Cao H., Huang Y., Xu H., Xie Z., Jiang Y., Wang X., Liu J. (2024). DNA Damage Response in Breast Cancer and Its Significant Role in Guiding Novel Precise Therapies. Biomark. Res..

[202] Raffoul J.J., Heydari A.R., Hillman G.G. (2012). DNA Repair and Cancer Therapy: Targeting APE1/Ref-1 Using Dietary Agents. J. Oncol..

[203] Garg P., Singhal G., Horne D., Salgia R., Singhal S.S. (2025). Metabolic Reprogramming in Breast Cancer: Pathways Driving Progression, Drug Resistance, and Emerging Therapeutics. Biochim. Biophys. Acta BBA Rev. Cancer.

[204] Choi E.J., Jang Y.Y., Choi E.J., Oh C.J. (2025). The Role of Lactate in Immune Regulation: A Metabolic Rheostat via Transporters, Receptors, and Epigenetic Modifiers. Cells.

[205] Liu S., Li Y., Yuan M., Song Q., Liu M. (2023). Correlation between the Warburg Effect and Progression of Triple-Negative Breast Cancer. Front. Oncol..

[206] Liu Y., He J., Chen J., Chen T., Li W., Yang Z., Zeng F. (2025). Programmed Cell Death in Triple-Negative Breast Cancer. Cell. Mol. Biol. Lett..

[207] Amaravadi R., Kimmelman A.C., White E. (2016). Recent Insights into the Function of Autophagy in Cancer. Genes Dev..

[208] Zhang H., Yue X., Chen Z., Liu C., Wu W., Zhang N., Liu Z., Yang L., Jiang Q., Cheng Q. (2023). Define Cancer-Associated Fibroblasts (CAFs) in the Tumor Microenvironment: New Opportunities in Cancer Immunotherapy and Advances in Clinical Trials. Mol. Cancer.

[209] Kzhyshkowska J., Shen J., Larionova I. (2024). Targeting of TAMs: Can We Be More Clever than Cancer Cells?. Cell. Mol. Immunol..

[210] Barkal A.A., Brewer R.E., Markovic M., Kowarsky M., Barkal S.A., Zaro B.W., Krishnan V., Hatakeyama J., Dorigo O., Barkal L.J. (2019). CD24 Signalling through Macrophage Siglec-10 Is a Target for Cancer Immunotherapy. Nature.

[211] Sami E., Paul B.T., Koziol J.A., ElShamy W.M. (2020). The Immunosuppressive Microenvironment in BRCA1-IRIS–Overexpressing TNBC Tumors Is Induced by Bidirectional Interaction with Tumor-Associated Macrophages. Cancer Res..

[212] Garofalo M., Villa A., Rizzi N., Kuryk L., Mazzaferro V., Ciana P. (2018). Systemic Administration and Targeted Delivery of Immunogenic Oncolytic Adenovirus Encapsulated in Extracellular Vesicles for Cancer Therapies. Viruses.

[213] Bai X., Ni J., Beretov J., Graham P., Li Y. (2021). Immunotherapy for Triple-Negative Breast Cancer: A Molecular Insight into the Microenvironment, Treatment, and Resistance. J. Natl. Cancer Cent..

[214] Xie Z., Fang Y., Zhang X., Fang Y., Li R., Guo Y., Yang Y., Yang S., Song L. (2025). The Emerging Role of Dendritic Cells in the Tumor Microenvironment: From Antigen Presentation to Targeted Immunotherapy. Cell Death Dis..

[215] Wang Y.-J., Fletcher R., Yu J., Zhang L. (2018). Immunogenic Effects of Chemotherapy-Induced Tumor Cell Death. Genes Dis..

[216] Choi Y., Kim S.A., Jung H., Kim E., Kim Y.K., Kim S., Kim J., Lee Y., Jo M.K., Woo J. (2024). Novel Insights into Paclitaxel’s Role on Tumor-Associated Macrophages in Enhancing PD-1 Blockade in Breast Cancer Treatment. J. Immunother. Cancer.

[217] Thomas R., Al-Khadairi G., Decock J. (2020). Immune Checkpoint Inhibitors in Triple Negative Breast Cancer Treatment: Promising Future Prospects. Front. Oncol..

[218] Dent R., Cortés J., Pusztai L., McArthur H., Kümmel S., Bergh J., Denkert C., Park Y.H., Hui R., Harbeck N. (2024). Neoadjuvant Pembrolizumab plus Chemotherapy/Adjuvant Pembrolizumab for Early-Stage Triple-Negative Breast Cancer: Quality-of-Life Results from the Randomized KEYNOTE-522 Study. JNCI J. Natl. Cancer Inst..

[219] Schmid P., Rugo H.S., Adams S., Schneeweiss A., Barrios C.H., Iwata H., Diéras V., Henschel V., Molinero L., Chui S.Y. (2020). Atezolizumab plus Nab-Paclitaxel as First-Line Treatment for Unresectable, Locally Advanced or Metastatic Triple-Negative Breast Cancer (IMpassion130): Updated Efficacy Results from a Randomised, Double-Blind, Placebo-Controlled, Phase 3 Trial. Lancet Oncol..

[220] Emens L.A., Adams S., Barrios C.H., Diéras V., Iwata H., Loi S., Rugo H.S., Schneeweiss A., Winer E.P., Patel S. (2021). First-Line Atezolizumab plus Nab-Paclitaxel for Unresectable, Locally Advanced, or Metastatic Triple-Negative Breast Cancer: IMpassion130 Final Overall Survival Analysis. Ann. Oncol..

[221] Miles D., Gligorov J., André F., Cameron D., Schneeweiss A., Barrios C., Xu B., Wardley A., Kaen D., Andrade L. (2021). Primary Results from IMpassion131, a Double-Blind, Placebo-Controlled, Randomised Phase III Trial of First-Line Paclitaxel with or without Atezolizumab for Unresectable Locally Advanced/Metastatic Triple-Negative Breast Cancer. Ann. Oncol..

[222] Cortes J., Cescon D.W., Rugo H.S., Nowecki Z., Im S.-A., Yusof M.M., Gallardo C., Lipatov O., Barrios C.H., Holgado E. (2020). Pembrolizumab plus Chemotherapy versus Placebo plus Chemotherapy for Previously Untreated Locally Recurrent Inoperable or Metastatic Triple-Negative Breast Cancer (KEYNOTE-355): A Randomised, Placebo-Controlled, Double-Blind, Phase 3 Clinical Trial. Lancet.

[223] Lin S., Fu B., Khan M. (2024). Identifying Subgroups Deriving the Most Benefit from PD-1 Checkpoint Inhibition plus Chemotherapy in Advanced Metastatic Triple-Negative Breast Cancer: A Systematic Review and Meta-Analysis. World J. Surg. Oncol..

[224] Nederlof I., Isaeva O.I., de Graaf M., Gielen R.C.A.M., Bakker N.A.M., Rolfes A.L., Garner H., Boeckx B., Traets J.J.H., Mandjes I.A.M. (2024). Neoadjuvant Nivolumab or Nivolumab plus Ipilimumab in Early-Stage Triple-Negative Breast Cancer: A Phase 2 Adaptive Trial. Nat. Med..

[225] Couch F.J., Hart S.N., Sharma P., Toland A.E., Wang X., Miron P., Olson J.E., Godwin A.K., Pankratz V.S., Olswold C. (2015). Inherited Mutations in 17 Breast Cancer Susceptibility Genes among a Large Triple-Negative Breast Cancer Cohort Unselected for Family History of Breast Cancer. J. Clin. Oncol..

[226] McCann K.E., Hurvitz S.A. (2018). Advances in the Use of PARP Inhibitor Therapy for Breast Cancer. Drugs Context.

[227] Jiao S., Xia W., Yamaguchi H., Wei Y., Chen M.-K., Hsu J.-M., Hsu J.L., Yu W.-H., Du Y., Lee H.-H. (2017). PARP Inhibitor Upregulates PD-L1 Expression and Enhances Cancer-Associated Immunosuppression. Clin. Cancer Res..

[228] Vinayak S., Tolaney S.M., Schwartzberg L., Mita M., McCann G., Tan A.R., Wahner-Hendrickson A.E., Forero A., Anders C., Wulf G.M. (2019). Open-Label Clinical Trial of Niraparib Combined with Pembrolizumab for Treatment of Advanced or Metastatic Triple-Negative Breast Cancer. JAMA Oncol..

[229] Tan T.J., Sammons S., Im Y.-H., She L., Mundy K., Bigelow R., Traina T.A., Anders C., Yeong J., Renzulli E. (2024). Phase II DORA Study of Olaparib with or without Durvalumab as a Chemotherapy-Free Maintenance Strategy in Platinum-Pretreated Advanced Triple-Negative Breast Cancer. Clin. Cancer Res..

[230] Singh D.D., Parveen A., Yadav D.K. (2021). Role of PARP in TNBC: Mechanism of Inhibition, Clinical Applications, and Resistance. Biomedicines.

[231] Hassan A., Aubel C. (2025). The PI3K/Akt/mTOR Signaling Pathway in Triple-Negative Breast Cancer: A Resistance Pathway and a Prime Target for Targeted Therapies. Cancers.

[232] Yu R., Zhu B., Chen D. (2022). Type I Interferon-Mediated Tumor Immunity and Its Role in Immunotherapy. Cell. Mol. Life Sci..

[233] Stopfer L., Rettko N.J., Leddy O., Mesfin J.M., Brown E., Winski S., Bryson B., Wells J.A., White F. (2022). MEK Inhibition Enhances Presentation of Targetable MHC-I Tumor Antigens in Mutant Melanomas. SSRN Electron. J..

[234] Kong S., Zhang J., Wang L., Li W., Guo H., Weng Q., He Q., Lou H., Ding L., Yang B. (2025). Mechanisms of Low MHC I Expression and Strategies for Targeting MHC I with Small Molecules in Cancer Immunotherapy. Cancer Lett..

[235] Yang B., Li X., Fu Y., Guo E., Ye Y., Li F., Liu S., Xiao R., Liu C., Lu F. (2021). MEK Inhibition Remodels the Immune Landscape of Mutant *KRAS* Tumors to Overcome Resistance to PARP and Immune Checkpoint Inhibitors. Cancer Res..

[236] Brufsky A., Kim S.B., Zvirbule Ž., Eniu A., Mebis J., Sohn J.H., Wongchenko M., Chohan S., Amin R., Yan Y. (2021). A Phase II Randomized Trial of Cobimetinib plus Chemotherapy, with or without Atezolizumab, as First-Line Treatment for Patients with Locally Advanced or Metastatic Triple-Negative Breast Cancer (COLET): Primary Analysis. Ann. Oncol..

[237] Liu Z., Li M., Jiang Z., Wang X. (2018). A Comprehensive Immunologic Portrait of Triple-Negative Breast Cancer. Transl. Oncol..

[238] Saleh R., Toor S.M., Khalaf S., Elkord E. (2019). Breast Cancer Cells and PD-1/PD-L1 Blockade Upregulate the Expression of PD-1, CTLA-4, TIM-3 and LAG-3 Immune Checkpoints in CD4+ T Cells. Vaccines.

[239] Xu B., Li Q., Zhang Q., Zhang Y., Ouyang Q., Zhang Y., Liu Q., Sun T., Xu J., Yang J. (2021). Abstract 1660: Preliminary Safety Tolerability & Efficacy Results of KN046 (an Anti-PD-L1/CTLA-4 Bispecific Antibody) in Combination with Nab-Paclitaxel in Patients with Metastatic Triple-Negative Breast Cancer (mTNBC). Cancer Res..

[240] Santa-Maria C.A., Kato T., Park J.-H., Flaum L.E., Jain S., Tellez C., Stein R.M., Shah A.N., Gross L., Uthe R. (2017). Durvalumab and Tremelimumab in Metastatic Breast Cancer (MBC): Immunotherapy and Immunopharmacogenomic Dynamics. J. Clin. Oncol..

[241] Famta P., Shah S., Dey B., Kumar K.C., Bagasariya D., Vambhurkar G., Pandey G., Sharma A., Srinivasarao D.A., Kumar R. (2025). Despicable Role of Epithelial–Mesenchymal Transition in Breast Cancer Metastasis: Exhibiting de Novo Restorative Regimens. Cancer Pathog. Ther..

[242] Cazet A.S., Hui M.N., Elsworth B.L., Wu S.Z., Roden D., Chan C.-L., Skhinas J.N., Collot R., Yang J., Harvey K. (2018). Targeting Stromal Remodeling and Cancer Stem Cell Plasticity Overcomes Chemoresistance in Triple Negative Breast Cancer. Nat. Commun..

[243] Brogna M.R., Varone V., DelSesto M., Ferrara G. (2025). The Role of CAFs in Therapeutic Resistance in Triple Negative Breast Cancer: An Emerging Challenge. Front. Mol. Biosci..

[244] Rebaudi F., De Franco F., Goda R., Obino V., Vita G., Baronti C., Iannone E., Pitto F., Massa B., Fenoglio D. (2024). The Landscape of Combining Immune Checkpoint Inhibitors with Novel Therapies: Secret Alliances against Breast Cancer. Cancer Treat. Rev..

